# Bacterial sensing: A putative amphipathic helix in RsiV is the switch for activating σ^V^ in response to lysozyme

**DOI:** 10.1371/journal.pgen.1007527

**Published:** 2018-07-18

**Authors:** Lincoln T. Lewerke, Paige J. Kies, Ute Müh, Craig D. Ellermeier

**Affiliations:** 1 Department of Microbiology and Immunology, Carver College of Medicine, University of Iowa, Iowa City, IA, United States of America; 2 Graduate Program in Genetics, University of Iowa, Iowa City, IA, United States of America; Max Planck Institute for Terrestrial Microbiology, GERMANY

## Abstract

Extra Cytoplasmic Function (ECF) σ factors are a diverse group of alternate σ factors bacteria use to respond to changes in the environment. The *Bacillus subtilis* ECF σ factor σ^V^ responds to lysozyme. In the absence of lysozyme, σ^V^ is held inactive by the anti-σ factor, RsiV. In the presence of lysozyme RsiV is degraded via regulated intramembrane proteolysis, which results in the release of σ^V^ and thus activation of lysozyme resistance genes. Signal peptidase is required to initiate degradation of RsiV. Previous work indicated that RsiV only becomes sensitive to signal peptidase upon direct binding to lysozyme. We have identified a unique domain of RsiV that is responsible for protecting RsiV from cleavage by signal peptidase in the absence of lysozyme. We provide evidence that this domain contains putative amphipathic helices. Disruption of the hydrophobic surface of these helices by introducing positively charged residues results in constitutive cleavage of RsiV by signal peptidase and thus constitutive σ^V^ activation. We provide further evidence that this domain contains amphipathic helices using a membrane-impermeable reagent. Finally, we show that upon lysozyme binding to RsiV, the hydrophobic face of the amphipathic helix becomes accessible to a membrane-impermeable reagent. Thus, we propose the amphipathic helices protect RsiV from cleavage in the absence of lysozyme. Additionally, we propose the amphipathic helices rearrange to form a suitable signal peptidase substrate upon binding of RsiV to lysozyme leading to the activation of σ^V^.

## Author summary

Signal transduction involves (i) sensing a signal, (ii) a molecular switch triggering a response, and (iii) altering gene expression. For *Bacillus subtilis’* response to lysozyme, we have a detailed understanding of (i) and (iii). Here we provide insights for a molecular switch that triggers the lysozyme response via σ^V^ activation. RsiV, an inhibitor of σ^V^ activity, is cleaved by signal peptidase only in the presence of lysozyme. Signal peptidase constitutively cleaves substrates that are translocated across the membrane. A domain-of-unknown-function (DUF4179) in RsiV contains the signal peptidase cleavage site, and protects RsiV from cleavage in the absence of lysozyme via amphipathic helices. In addition to RsiV, DUF4179 is found in an unrelated and uncharacterized anti-σ factor present in Firmicutes including within some clinically-relevant species.

## Introduction

One key survival feature of all living organisms is the ability to recognize changes in their environments and respond appropriately. One signal transduction system bacteria use to sense and respond to environmental stresses are Extracytoplasmic Function (ECF) sigma (σ) factors [1,2]. ECF σ factors are a diverse family of alternative σ factors that are responsible for transcribing a wide variety of genes in response to environmental signals, often extracellular stress [3,4]. One hallmark of ECF σ factors is the presence of an anti-σ factor that is responsible for sequestering σ activity in the absence of signal. When bacteria encounter a specific signal, the anti-σ factor is inactivated via one of three mechanisms: 1) degradation of the anti-σ factor [3–5] 2) conformational change of the anti-σ factor [6–9] or 3) a partner switching mechanism in which an anti-anti-σ factor binds the anti-σ factor [10–13]. All three mechanisms result in the signal-dependent release of the σ factor and subsequent transcription of genes involved with the stress response.

Degradation of the anti-σ factor is frequently achieved by Regulated Intramembrane Proteolysis (RIP) [4,14,15], a stepwise proteolytic cascade. In most cases, cleavage occurs extracellularly at site-1 followed by an intramembrane site-2 protease. Cytosolic proteases degrade the remaining portion of the protein, releasing the σ factor to interact with RNA polymerase [4,14,15]. Cleavage at site-1 is typically the rate-limiting step of this cascade and thus is the regulated step of ECF σ factor activation [16–19]. A well-studied example of such regulation is the ECF σ factor σ^E^ in *E*. *coli* which is responsible for responding to cell envelope stress and is held inactive by the membrane bound anti-σ factor RseA [18,20]. Unfolded outer membrane proteins bind to the PDZ domain of DegS leading to the activation of DegS and cleavage of RseA at site-1 [21]. This cleavage event initiates the RIP cascade where RseA is further degraded by the intramembrane protease RseP and cytosolic proteases resulting in the activation of σ^E^ [14,19,22]. The *B*. *subtilis* ECF σ factor σ^W^ is another example where the rate-limiting step of anti-σ factor inactivation is proteolytic cleavage at site-1. RsiW is the anti-σ factor that holds σ^W^ in an inactive state in the absence of signal [17]. When cell envelope stresses are present, the site-1 protease PrsW cleaves RsiW and initiates a RIP cascade, resulting in the activation of σ^W^ [23,24].

In *B*. *subtilis* the ECF σ factor σ^V^ belongs to the ECF30 subfamily which is found primarily in low GC gram positive Firmicutes including *C*. *difficile* and *E*. *faecalis* [1]. σ^V^ is held inactive by its anti-σ factor RsiV and the inducing signal is C-type lysozyme [25]. Lysozyme is a component of the innate immune system that degrades peptidoglycan by cleaving β-(1,4)-linked *N*-acetylmuramic acid and *N*-acetylglucosamine [26–28]. σ^V^ is responsible for transcribing genes involved in lysozyme resistance including *oatA* which encodes an O-acetylase that adds an acetyl group to peptidoglycan increasing resistance to lysozyme cleavage [16,29–31]. The *dltABCDE* operon is also transcribed by σ^V^ and encodes genes responsible for modifying the charge of teichoic acid which presumably works to repel positively charged lysozyme [25,31–34]. In the absence of σ^V^ both *C*. *difficile* and *E*. *faecalis* become more sensitive to lysozyme [35,36].

**Fig 1:**
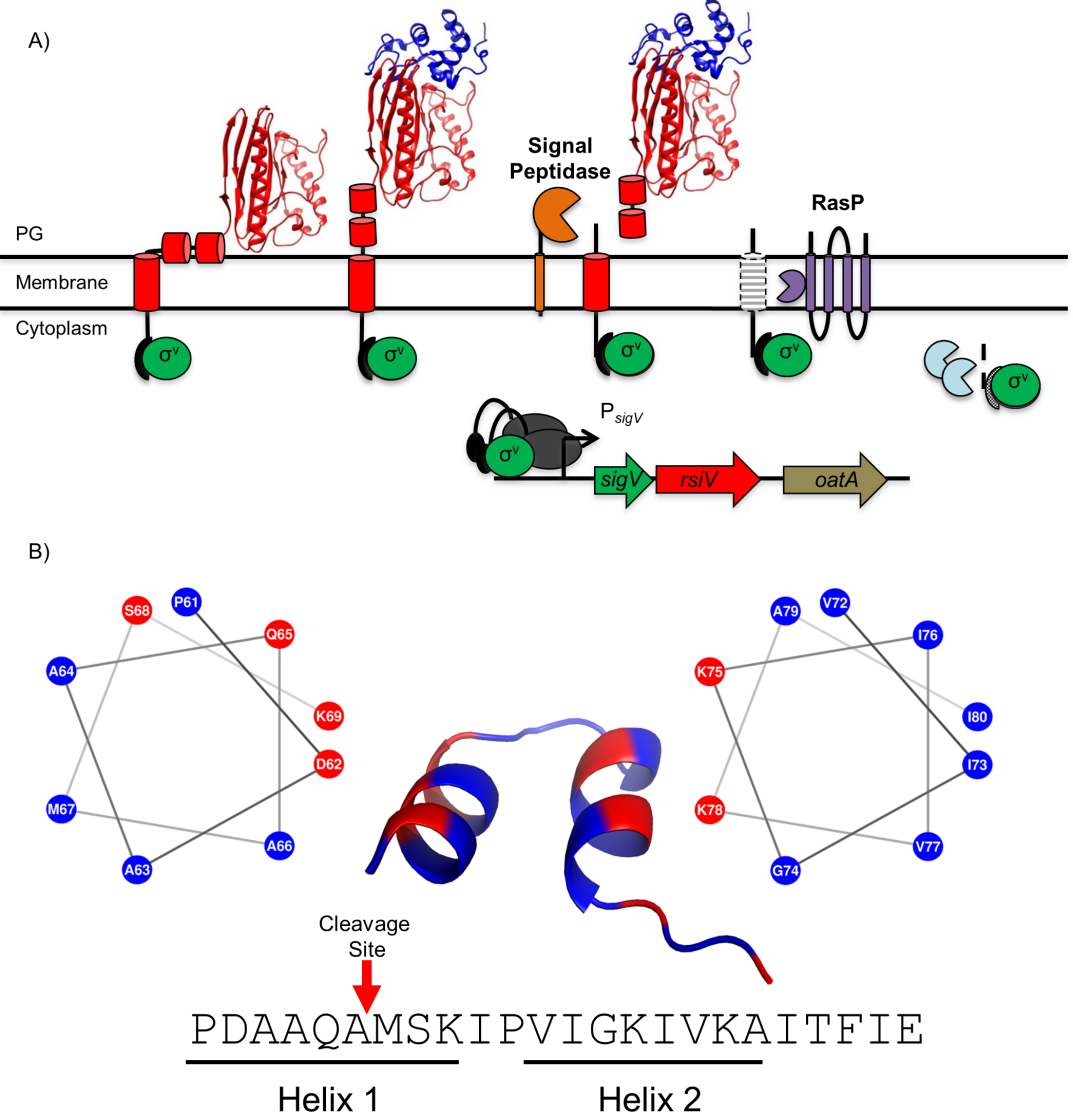
Model of σ^V^ activation. **A.** The σ factor σ^V^ is shown in green, the anti-σ factor RsiV is shown in red with cartoon cylinders representing the unsolved residues 1–75. Signal peptidase (Sip) is shown in yellow, the site two protease RasP is shown in purple and lysozyme is shown in blue. In the absence of lysozyme, RsiV is resistant to signal peptidase cleavage via the interaction of two amphipathic helices with the membrane that restrict access to the cleavage site. In this state RsiV sequesters σ^V^ activity and prevents transcription of σ^V^-dependent genes. Once RsiV binds lysozyme, the amphipathic helices are forced out of the membrane and signal peptidase is allowed access to the cleavage site leading to site-1 degradation of RsiV. The remaining transmembrane portion of RsiV is further degraded by the site-2 protease RasP, after which RsiV is further degraded by cytosolic proteases. This allows σ^V^ to interact with RNA polymerase (Grey) and promote transcription of σ^V^-dependent genes. **B.** An set of 203 RsiV homologs were aligned using Clustal Omega [70] and the alignment was used with weblogo [44] to generate a sequence logo (S1 Fig). The corresponding sequence in *B*. *subtilis* was run through the secondary structure prediction software PEP-FOLD-3 [45] and the results are shown by the cartoon structure with two α-helices. Each helix was modeled with a helical wheel generator [47] and labeled below the sequence designated as Helix 1 (Left) and Helix 2 (Right). Hydrophobic amino acids are represented in blue and hydrophilic residues are represented in red. The cleavage site of RsiV is represented with a red arrow. This bioinformatic analysis suggests the presence of two conserved amphipathic helices directly after the transmembrane domain of RsiV that function to protect RsiV from site-1 cleavage by signal peptidase.

Activation of σ^V^ occurs through RIP-mediated degradation of the anti-σ factor RsiV and occurs only in the presence of C-type lysozyme [16,37,38]. The current model of σ^V^ activation is shown in Fig 1A. Our lab has previously shown that signal peptidase is the site-1 protease responsible for removing the extracellular portion of RsiV [38]. After site-1 cleavage, the remaining transmembrane portion of RsiV is then degraded constitutively by the intramembrane metalloprotease RasP at site-2. The remaining cytoplasmic portion of RsiV is presumably degraded by cytosolic proteases [16]. RIP-mediated cleavage of RsiV leads to the release of σ^V^ and subsequent transcription of σ^V^-controlled genes resulting in increased lysozyme resistance for the bacterium.

In bacteria, signal peptidases are associated with the secretion machinery and are critical for cell function [39,40]. Signal peptidases are typically responsible for cleaving secreted pre-proteins containing a signal peptide, thus releasing them from the membrane. Signal peptidase function is not known to be regulated and cleavage is believed to occur during or shortly after translocation [39–41]. The findings in *B*. *subtilis* then raise interesting questions: how is RsiV able to resist signal peptidase cleavage in the absence of lysozyme? What changes occur once lysozyme binds to create a suitable substrate for signal peptidase?

Here we provide evidence for the presence of amphipathic helices in RsiV that protect it from signal peptidase cleavage in the absence of lysozyme. We identified the region required to protect fusion proteins from signal peptidase cleavage. We determined that disruption of the putative amphipathic helices by introducing lysine residues results in constitutive cleavage by signal peptidase. We show using a membrane-impermeable reagent that the hydrophobic face of the amphipathic helixes can only be labeled in the presence of lysozyme. We also propose a model to explain how RsiV and lysozyme binding leads to a suitable signal peptidase substrate.

## Results

### RsiV residues 1–86 are sufficient for protection of a membrane-targeted GFP from signal peptidase cleavage

**Fig 2:**
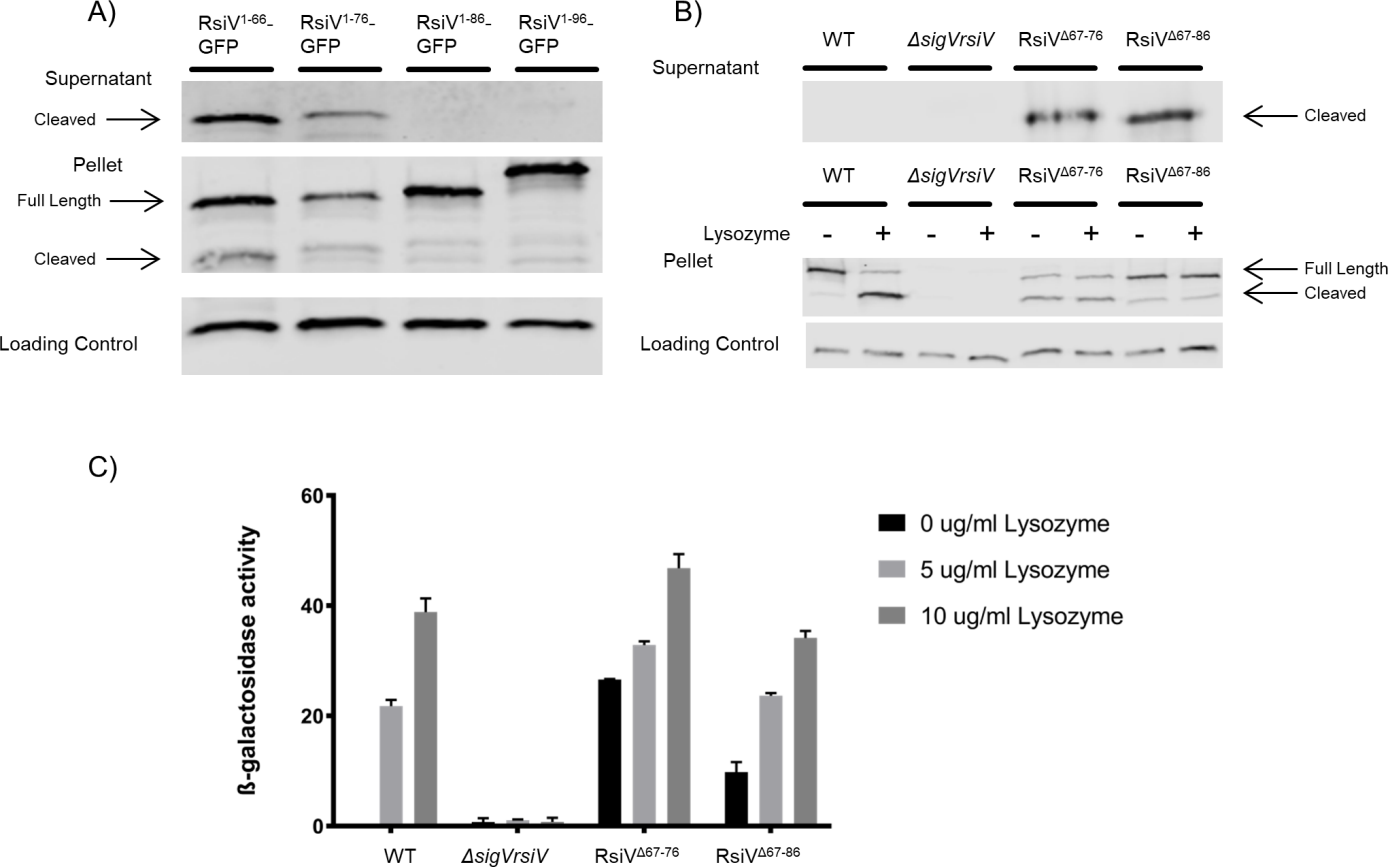
Amino acids after the cleavage site are involved in the protection of RsiV from signal peptidase cleavage. **A.** Cells producing various lengths of N-terminal RsiV (1–66, 1–76, 1–86 and 1–96) fused to GFP were grown to mid log. Cell pellets and the supernatants were collected and analyzed by western blot with α-RsiV^59-258^ antibodies. Streptavidin IR680LT was used detect PycA which served as a loading control [67]. The color blot showing both pellet and loading control on a single gel is S2 Fig. **B.** Deletions in RsiV after the cleavage site were created (RsiV^Δ67–76^, RsiV^Δ67–86^) and expressed under an IPTG inducible promoter. The supernatants were collected. The cells were then exposed to -/+ lysozyme (10μg/mL). Samples were analyzed by western blot with α-RsiV^59-258^ antibodies and streptavidin IR680LT was used to detect PycA as a loading control [67]. The color blot showing both pellet and loading control on a single gel is S3 Fig. **C.** The deletion constructs (RsiV^Δ67–76^, RsiV^Δ67–86^) were created in a strain that carries a P_*sigV*_*-lacZ* reporter. σ^V^ activity was determined by measuring β-galactosidase activity. This experiment was done in triplicate and standard deviation is represented by error bars.

We previously demonstrated that RsiV is proteolytically degraded in a RIP-dependent manner in the presence of lysozyme leading to the activation of σ^V^ [16,37]. The protease required for site-1 cleavage of RsiV is signal peptidase [37,38]. However signal peptidase is only able to efficiently cleave RsiV in the presence of lysozyme [38]. It has been proposed that in the absence of lysozyme, RsiV is in a conformation that is resistant to signal peptidase cleavage. Upon binding to lysozyme, RsiV is hypothesized to undergo a conformational change that allows signal peptidase access to the cleavage site, leading to degradation of RsiV and subsequent σ^V^ activation [42]. Site-1 cleavage of RsiV by signal peptidase occurs between residues 66 and 67 [37]. To identify the key residues of RsiV sufficient for protection from signal peptidase, we fused the signal peptide of RsiV (amino acids 1–66) to GFP and expressed the fusion protein in *B*. *subtilis*. We predict that if the RsiV signal peptide is able to protect from signal peptidase cleavage, then the fusion of these residues to GFP would be sufficient to block secretion of GFP into the culture supernatant. We analyzed the RsiV^1-66^-GFP fusion by performing western blot analysis of the whole cells and concentrated culture supernatant using an anti-GFP antibody. We detected cleaved GFP in the culture supernatants from strains producing RsiV^1-66^-GFP suggesting the signal peptide alone is not sufficient to protect from signal peptidase (Fig 2A).

We then created constructs with various lengths of the RsiV N-terminus fused to GFP and expressed in *B*. *subtilis* to determine if additional residues after the signal peptide (RsiV^1-76^-GFP, RsiV^1-86^-GFP, and RsiV^1-96^-GFP) are sufficient to protect GFP from cleavage by signal peptidase. Cells producing these proteins were analyzed by western blot as described above. We found that cleaved GFP was present in the culture supernatant of strains producing RsiV^1-66^-GFP and RsiV^1-76^-GFP (Fig 2A). However, we did not detect a cleaved GFP band in the supernatant for RsiV^1-86^-GFP, or RsiV^1-96^-GFP. Importantly, we were able to detect uncleaved RsiV-GFP in whole cells confirming they were produced at similar levels (Fig 2A). These results suggest that residues 1–86 of RsiV are sufficient to protect from signal peptidase cleavage.

### Residues after the cleavage site are required to protect RsiV from signal peptidase cleavage

Our data suggest that residues 1–86 are sufficient to protect the RsiV-GFP fusion proteins from signal peptidase cleavage, but residues 1–76 were not sufficient. To determine if the region after the signal peptidase cleavage site is required to protect RsiV from signal peptidase cleavage we constructed internal deletions of RsiV after the signal peptidase cleavage site; RsiV^Δ67–76^ and RsiV^Δ67–86^. To monitor signal peptidase cleavage of RsiV in the presence and absence of lysozyme, the cell pellets and culture supernatants were collected and analyzed by western blotting with anti-RsiV antibodies. Similar to the previous assay, if a cleavage product is detected in the supernatant it indicates that RsiV is being cleaved in the absence of lysozyme. Consistent with previous observations, wild-type (WT) RsiV was cleaved only in the presence of lysozyme (Fig 2B) [37]. In contrast for both deletion constructs a cleavage product was detected in the culture supernatant in the absence of lysozyme (Fig 2B).

We constructed the same deletions in a reporter strain to measure their impact on σ^V^ activity. We measured σ^V^ activity using a P_*sigV*_*-lacZ* promoter fusion which is dependent upon σ^V^ for expression [25,31]. We found, as expected, that RsiV^Δ67–76^ and RsiV^Δ67–86^ had increased basal levels of P_*sigV*_*-lacZ* expression indicating increased σ^V^ activation (Fig 2C). In addition, P_*sigV*_*-lacZ* was induced only ~2-fold by lysozyme when the region located after the cleavage site was deleted (Fig 2C). We also performed a western blot of the pellet and supernatant from the strains used in the σ^V^ activity assay and found similar results to IPTG expression strains (S4 Fig). WT RsiV was not cleaved while both RsiV deletion constructs were degraded in the absence of lysozyme (S4 Fig). Taken together, these results suggest that residues within 67–86 are required for the protection of RsiV from signal peptidase cleavage in the absence of lysozyme. We propose that residues surrounding the cleavage site of RsiV to amino acid 86 contain a region that is important for blocking signal peptidase cleavage of RsiV in the absence of lysozyme.

### The protective region of RsiV is highly conserved among homologs

Based on our observation that residues 67–86 are necessary to protect against signal peptidase cleavage, we sought to characterize the region that is responsible for resistance to signal peptidase. An alignment of 203 RsiV homologs was used to create a sequence logo showing a high degree of homology for residues 61–84 (S1 Fig) [37,43,44]. The sequence logo also revealed an alternating pattern of hydrophobic and hydrophilic residues suggesting the presence of a potential amphipathic helix (Fig 1B). We used the secondary structure prediction software PEP-FOLD3 to predict the structure of this region in *B*. *subtilis* and we identified a predicted structure with two α-helices separated by a loop (Fig 1B) [45,46]. Upon investigation of the predicted structure, we identified a hydrophobic and hydrophilic face for each helix suggesting the predicted secondary structure contains two amphipathic helices. Helical wheel projections of each helix were created using NetWheels [47] and further suggest the presence of putative amphipathic helices containing both a hydrophobic and hydrophilic face (Fig 1B). The putative amphipathic helices are part of a domain of unknown function DUF4179 [48]. In the case of another DUF4179 member, the anti-σ factor BAS1627 from *Bacillus anthracis* the structure was solved (3FBQ) [49] revealing two α-helices separated by a turn much like our predicted structure (Fig 1B). Analysis of Pfam database revealed that the DUF4179 domain is largely restricted to the phyla Firmicutes (Table 1). RsiV and BAS1627 are members of the ECF30 family which are almost exclusively found in Firmicutes (48) and it appears the DUF4179 is restricted to these anti-sigma factor families.

**Table 1:** Phylum distribution of DUF4179*.

Phylum	Number of Species	Number of Sequences
Firmicutes	203	702
Actinobacteria	10	14
Chloroflexi	2	3
Mucoromycota	1	1
Streptophyta	1	2

*as of June 27, 2018

### Disruption of hydrophobic residues in the potential amphipathic helix leads to constitutive σ^V^ activation and RsiV degradation

**Fig 3:**
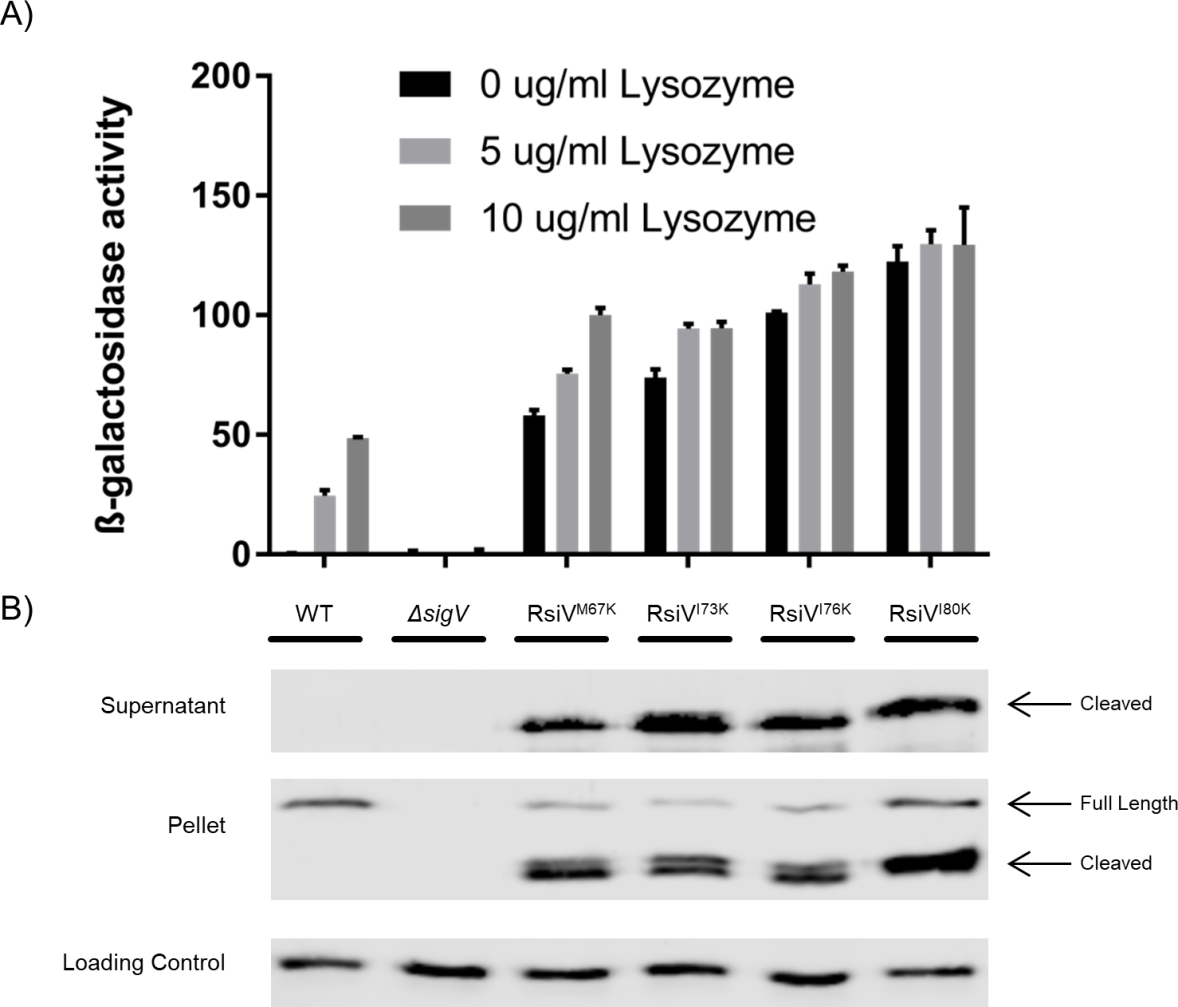
Lysine substitutions in the amphipathic helices lead to constitutive σ^V^ activity. **A**. Various hydrophobic residues of the amphipathic helices were substituted with lysine residues to disrupt the charge of the amphipathic helix (M67K, I73K, I76K, I80K). These constructs were measured for σ^V^ activity using a P_*sigV*_*-lacZ* reporter and a β-galactosidase assay. This experiment was done in triplicate and standard deviation is represented by error bars. **B.** The lysine substitution constructs (M67K, I73K, I76K, I80K) were further analyzed by western blot to measure RsiV degradation. Cells were grown to mid log. The pellet and supernatants were collected and samples were analyzed by western blot with α-RsiV^59-258^ antibodies. Streptavidin IR680LT was used detect PycA which served as a loading control [67]. The color blot showing both pellet and loading control on a single gel is S5 Fig.

Our *in-silico* analysis of RsiV revealed potential amphipathic helices that we hypothesized could be responsible for blocking signal peptidase cleavage in the absence of lysozyme. To test this hypothesis, we sought to disrupt the amphipathic helices by introducing a positive charge into the hydrophobic face. We constructed strains in which a hydrophobic residue of Helix 1 (M67), Helix 2 (I73, I76), or a residue directly after the predicted amphipathic helices (I80) were each changed to a lysine residue. The resulting constructs were analyzed for σ^V^ activity using the P_*sigV*_*-lacZ* reporter. We found that a lysine substitution at each hydrophobic residue resulted in dramatic (>100-fold) increase in the basal level of P_*sigV*_*-lacZ* expression in the absence of lysozyme (Fig 3A). When incubated with lysozyme (10 μg/ml), cells producing RsiV^M67K^ showed only a modest ~2-fold increase while the remaining mutants showed no further increase (Fig 3A). Thus, changing select hydrophobic residues to positively charged lysine residues results in constitutive σ^V^ activity which suggests that RsiV is being cleaved by signal peptidase in the absence of lysozyme.

To ensure RsiV was being produced and to determine if it was being cleaved at site-1 by signal peptidase in the absence of lysozyme we measured RsiV levels both in the cells and in the culture supernatant by western blot using anti-RsiV antibodies. In each case we observed cleaved RsiV in the culture supernatants of cells producing RsiV with a lysine substitution but not WT RsiV (Fig 3B). In addition, we observed increased levels of RsiV in whole cell fractions of the cells producing RsiV with lysine substitutions (Fig 3B). This is likely due to increased activation of σ^V^ and thus increased expression of *rsiV* itself. These results suggest that changing the hydrophobic residues of these putative amphipathic helices to a positively charged residue leads to increased cleavage of RsiV in the absence of lysozyme.

To ensure that these mutations did not simply lead to misfolded RsiV, we used Circular Dichroism spectroscopy (CD) to compare the secondary structure of RsiV^A66W I76K^ and RsiV^A66W I80K^ to WT RsiV and RsiV^A66W^. The addition of RsiV^A66W^ was necessary to allow for purification of the proteins without cleavage by signal peptidase. CD spectra showed troughs at 208 and 222 nm indicating helical secondary structure in the protein. Moreover, the CD spectra of all the variants are very similar to WT, suggesting that the lysine mutations do not result in protein with any gross change in secondary structure (S6 Fig). This supports a model in which an amphipathic helix protects RsiV cleavage in the absence of lysozyme and disruption of the amphipathic nature of these helices results in lysozyme independent cleavage of RsiV by signal peptidase.

### Hydrophobic residues of the amphipathic helices are differentially labeled dependent on lysozyme

**Fig 4:**
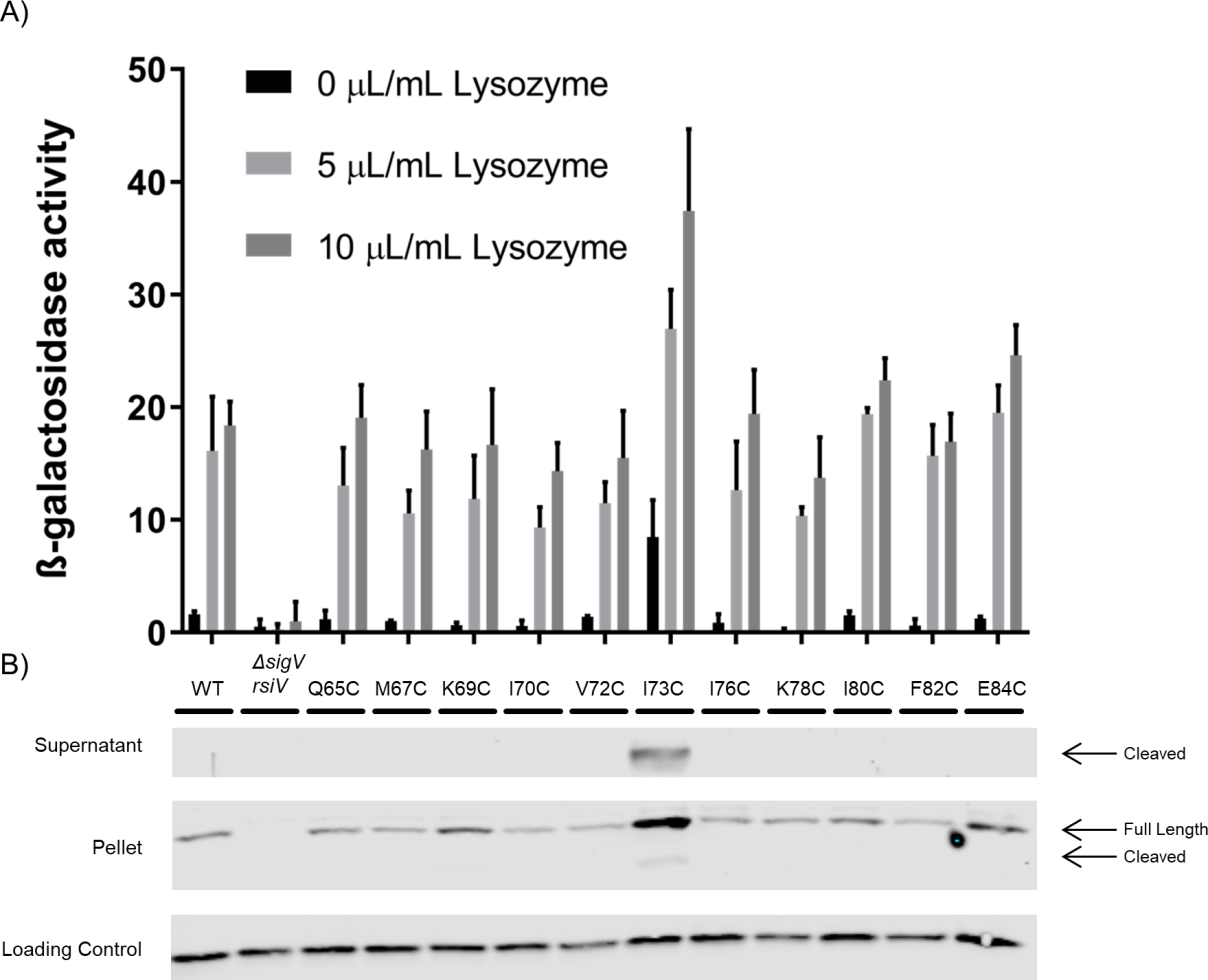
Cysteine substitutions do not affect RsiV function. **A.** Various cysteine substitution in the amphipathic helices of RsiV were created (Q65C, M67C, K69C, I70C, V72C, I73C, I76C, K78C, I80C, F82C, E84C) to determine the effect on σ^V^ activity using a P_*sigV*_*-lacZ* reporter and a β-galactosidase assay. This experiment was done in triplicate and standard deviation is represented by error bars. **B.** The cysteine substitutions were further analyzed by western blot to measure RsiV degradation. Cells were grown to mid log. The pellet and supernatants were collected and samples were analyzed by western blot with α-RsiV^59-258^ antibodies. Streptavidin IR680LT was used detect PycA which served as a loading control [67]. The color blot showing both pellet and loading control on a single gel is S7 Fig.

We hypothesize the hydrophobic face of the amphipathic helices are interacting with the cell membrane and this interaction is limiting signal peptidase access to the cleavage site. To test whether the hydrophobic residues are membrane embedded we used a Substituted Cysteine Accessibility Method (SCAM) assay which takes advantage of the fact that RsiV lacks endogenous cysteine residues. Before performing the SCAM assay, we first tested the effect of the various RsiV cysteine substitutions on σ^V^ activity by monitoring expression of the P_*sigV*_*-lacZ* promoter fusion in response to increasing concentrations of lysozyme. We found that the majority of cysteine substitutions did not alter activation of σ^V^ in response to lysozyme and behaved like WT RsiV (Fig 4A). Only one cysteine substitution (RsiV^I73C^) led to increased σ^V^ activity in the absence of lysozyme (4A). Additionally, we monitored RsiV degradation by testing both the pellet and supernatant of these constructs for RsiV by western blot analysis. Similar to the σ^V^ activity assay, only RsiV^I73C^ was cleaved in the absence of lysozyme (Fig 4B). These results suggest that single cysteine substitutions of RsiV, except for RsiV^I73C^, do not impact σ^V^ activity and result in a functional RsiV.

**Fig 5:**
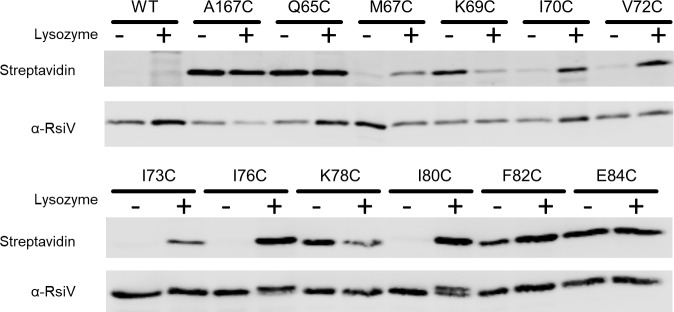
Hydrophobic residues are differentially labeled in lysozyme conditions. To allow affinity purification, RsiV was expressed with an N-terminal 6xHis-tag which does not alter RsiV function. An A66W mutation was introduced to prevent cleavage when incubated in the presence of lysozyme. Various residues (Q65, M67, K69, I70, V72, I73, I76, K78, I80, F82, E84) were mutated to a cysteine to probe their accessibility to a membrane-impermeable dye A167C was created as a positive control as it is located in the lysozyme binding pocket and expected to be outside of the membrane. Cells were grown to mid log and the SCAM assay was performed. The resulting purified products were analyzed by western blot with Streptavidin IR680LT to detect the MPB label and α-RsiV^59-258^ antibodies to serve as a loading control.

To perform the SCAM assay we constructed a strain producing 6xHis-RsiV^A66W^. The 6xHis-tag was added to allow for purification of RsiV mutant proteins after labeling. The A66W mutant was necessary to allow for purification of RsiV after lysozyme addition as we previously demonstrated that RsiV^A66W^ blocks site-1 cleavage [37,38]. We then introduced cysteine substitutions at various positions along the putative amphipathic helices of RsiV (Fig 1B). To label cysteine substitutions of 6xHis-RsiV^A66W^ we used (Na-(3-maleimidylpropionyl) biocytin (MPB) [50,51]. MPB is a membrane-impermeable molecule with a biotin group that allows for detection using streptavidin conjugates and a maleimide group capable of covalently binding to cysteine [51,52]. Cells were either exposed to lysozyme (150 μg/ml) or left untreated. MPB (50 μM) was added to label extracellular cysteines. Next, RsiV was affinity purified using nickel resin. We then performed western blotting to analyze RsiV levels with anti-RsiV and MPB labeled RsiV was detected using Streptavidin IR680LT. As a negative control for MPB we included RsiV^A66W^ which lacks cysteines and found that, as expected, we could not detect biotinylated RsiV. As a positive control, we introduced a cysteine at position 167 which is where RsiV interacts with lysozyme and we expect to be present extracellularly [42]. We found that RsiV^A66W A167C^ was labeled when incubated in the presence and absence of lysozyme (Fig 5).

We observed that hydrophilic residues (Q65C, K69C, K78C, and E84C) could be labeled in both the presence and absence of lysozyme indicating these residues are outside of the membrane and accessible to label (Fig 5). Conversely, hydrophobic residues (M67C, I70C, V72C, I73C, I76C, and I80C) do not label in the absence of lysozyme suggesting they are embedded in the membrane when lysozyme is not present. In the presence of lysozyme these same hydrophobic residues are now labeled, indicating they are accessible to the label. F82C is a hydrophobic residue that labels in both the absence and presence of lysozyme indicating that it is not embedded in the membrane (Fig 5). Interestingly, F82 is not predicted to be part of the α-helices (Fig 1B) and was previously observed to be part of the β-sheet in the RsiV-Lysozyme co-structure [42]. These results further suggest the presence of amphipathic helices that protect RsiV from signal peptidase cleavage in the absence of lysozyme.

### Disruption of the putative amphipathic helices forces hydrophobic residues out of the membrane

**Fig 6:**
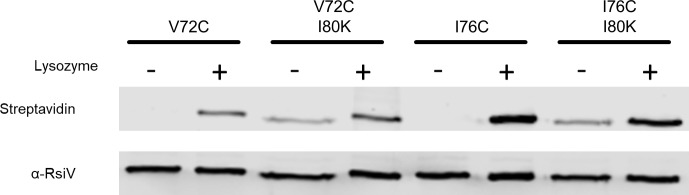
Lysine substitution disrupts the membrane association of hydrophobic residues. To determine the effect of lysine substitution on hydrophobic residue accessibility in the amphipathic helices we created a construct with N-term 6xHis to purify RsiV and A66W to allow for the purification of RsiV exposed to lysozyme. The cysteine mutations (V72C and I76C) were combined with an I80K substitution to create the constructs RsiV^V72C I80K^ and RsiV^I76C I80K^. Cells were grown to mid log and the SCAM assay was performed. The resulting purified products were analyzed by western blot with Streptavidin IR680LT to detect the MPB label and α-RsiV^59-258^ antibodies to serve as a loading control.

We have shown that changing hydrophobic residues of the amphipathic helix to lysine leads to constitutive cleavage of RsiV and that cysteine substitutions in the hydrophobic face are not accessible in the absence of lysozyme. To measure the effects of the lysine mutations on accessibility of the hydrophobic face we combined I80K with different hydrophobic cysteine substitutions (V72C, I76C) and analyzed these strains using the SCAM assay in the presence and absence of lysozyme. As we showed previously RsiV^V72C^ and RsiV^I76C^ did not label in absence of lysozyme and upon the addition of lysozyme they were labeled by MPB (Fig 6). Interestingly, the cysteines in RsiV^V72C, I80K^ and RsiV^I76C, I80K^ were accessible to MPB labelling in both the presence and absence of lysozyme (Fig 6). This suggests the hydrophobic face is no longer in the membrane when there is a lysine substitution. This result is consistent with the model of lysine substitutions in the hydrophobic face of the amphipathic helix displacing the helix from the membrane and leading to constitutive cleavage of RsiV and thus constitutive σ^V^ activation.

## Discussion

Here we describe a mechanism by which RsiV resists signal peptidase cleavage in the absence of lysozyme and propose a model for how lysozyme binding to RsiV leads to degradation of the anti-σ factor and subsequent activation of σ^V^. We propose that RsiV contains amphipathic helices immediately after the transmembrane domain that interact with the membrane and restrict signal peptidase access to the cleavage site, leading to a stable protein in the absence of signal. This is supported by: 1) this region is necessary and sufficient to protect from signal peptidase cleavage 2) disruption of hydrophobic residues by lysine substitution leads to constitutive activation of σ^V^ and 3) hydrophobic residues remain unlabeled in the absence of lysozyme. We also propose that lysozyme binding to RsiV leads to a conformational change that drives the amphipathic helices from the membrane, allowing signal peptidase to access the cleavage site. This model is supported by the differential labeling of residues on the hydrophobic face of the amphipathic helix.

### Putative amphipathic helices provide resistance from signal peptidase cleavage

Our data suggests the presence of amphipathic helices that are required for the protection of RsiV from signal peptidase cleavage. The fusion construct RsiV^1-86^-GFP demonstrates that this region is sufficient to block signal peptidase cleavage, whereas fewer residues fail to protect. Deletions of this region lead to both constitutive cleavage of RsiV and constitutive σ^V^ activity showing that this region is necessary to resist signal peptidase cleavage. This study also found that disrupting the hydrophobicity of the amphipathic helices through lysine substitution abolished protection from signal peptidase cleavage. The sensitivity to signal peptidase cleavage is likely due to the lysine substitutions forcing the helices out of the membrane and is supported by the observation that a lysine substitution leads to accessibility of the hydrophobic face in the absence of lysozyme.

The results of the cysteine labeling assay show that in the absence of lysozyme, residues on the hydrophobic face of the amphipathic helix are inaccessible to label. This suggests these helices are either membrane-associated, interacting with each other via the hydrophobic faces, or the hydrophobic residues are buried inside of RsiV. Our observation that RsiV^1-86^-GFP, which lacks the majority of extracellular RsiV, yet is protected from signal peptidase cleavage implies that the amphipathic helices are not interacting with RsiV. Thus, we favor a model where they interact with the membrane or with one another via their hydrophobic surfaces thus protecting them from signal peptidase cleavage.

### Lysozyme binding drives amphipathic helices out of the membrane

Our lab has previously shown that lysozyme binds to RsiV and we have obtained the crystal structure of this complex [42]. We have also shown that the binding of lysozyme to RsiV is necessary for signal peptidase to cleave RsiV [37,38,42]. Previously it has been hypothesized that the binding of lysozyme to RsiV induces a conformation change leading to the susceptibility of signal peptidase cleavage. We propose that binding of lysozyme to RsiV causes the amphipathic helices to no longer interact with the membrane, allowing signal peptidase access to the previously sequestered signal peptide cleavage site. This model is supported by the observation that residues which we presume to interact with membrane in the absence of lysozyme become accessible when lysozyme binds to RsiV.

One major question remains: what drives α-helices from the membrane upon lysozyme binding? Signal peptidases preferentially cleave flexible and accessible regions of a protein over ordered structures such as α-helices [53]. The co-structure of RsiV and lysozyme shows that residues 79–89 are in a β-sheet while residues 59–78 are disordered when bound to lysozyme [42]. In the absence of lysozyme, however, residue 80 is unable to be labeled by a membrane-impermeable dye, suggesting it is membrane embedded. The latter is supported by structural prediction programs which propose amphipathic helices for region 61–79. Thus, one possible mechanism responsible for driving the amphipathic helices from the membrane is that lysozyme binding causes part of the helix to convert to a β-sheet. A structure of RsiV alone is needed to answer these questions.

### Additional examples of amphipathic helices controlling degradation

There are other reports of amphipathic helices controlling degradation or cleavage of proteins by signal peptidases. One example is the HIV glycoprotein 160 (gp160) which is processed in the endoplasmic reticulum of infected cells and forms the soluble subunit gp120 which is involved in viral entry [54,55]. Once gp160 is translocated across the ER membrane, an α-helix surrounding the signal peptidase cleavage site interacts with the ER membrane and prevents cleavage until the protein is properly folded [56]. This α-helix works as a quality control mechanism to ensure proper folding prior to signal peptidase cleavage. In fact when the α-helix is disrupted, proper folding does not occur [56].

Another example of an amphipathic helix controlling degradation is the human protein squalene monooxygenase (SM) which is the second, rate-limiting step in cholesterol biosynthesis [57]. When cholesterol levels are low, the amphipathic helix of SM is associated with the ER membrane and SM is able to function normally. Once cholesterol levels rise, the amphipathic helix is disassociated from the membrane likely due to cholesterol thickening and flattening of the ER membrane. This disassociation leads to the degradation of SM by the ubiquitin–proteasome system and leads to a decrease in cholesterol biosynthesis [58]. These systems are similar to RsiV in that they all use a membrane-associated amphipathic helix to control the access of a protease to its cleavage site resulting in blocking or delaying cleavage. These observations may suggest a broader use of amphipathic helices in controlling the cleavage of other as yet unidentified signal peptidase substrates.

### DUF4179 contains putative amphipathic helices

The transmembrane segment and putative amphipathic helices of RsiV are part of a domain of unknown function, DUF4179 [48]. This domain is found primarily in Firmicutes and a crystal structure has been solved for a protein containing this domain. The crystal structure (3FBQ) is the extracellular domain of an ECF anti-σ factor from *B*. *anthracis* [49]. The structure shows a region directly after the transmembrane domain that has two short α-helices similar to the predicted structure of the region surrounding the RsiV cleavage site. However, the rest of the protein is unrelated to RsiV. This structural information, combined with the results of our study, and the role of amphipathic helices controlling signal peptidase cleavage in other organisms raises the intriguing possibility that this could be a common motif in ECF signaling, playing an important role in protecting the anti-σ factor from degradation, and triggering the proteolytic cascade that releases the cognate σ-factor in the presence of the appropriate signal.

## Materials and methods

### Strain construction

All *B*. *subtilis* strains (Table 2) are isogenic derivatives of PY79, a prototrophic derivative of *B*. *subtilis* strain 168 [59]. Plasmids used in this study are listed in Table 3 and were confirmed by DNA sequencing (Iowa State DNA Sequencing Facility). *B*. *subtilis* strains were transformed using a one-step method to induce competence previously described by [60]. Plasmids used for transformation into *B*. *subtilis* were first created using site directed mutagenesis (Supplemental Methods) along with isothermal assembly [61] and propagated in *E*. *coli* Omnimax cells. Oligonucleotide primers used for PCR and site-directed mutagenesis are listed in S1 Table.

**Table 2:** Strains list.

Strains	Genotype	Reference
*E*. *coli* strains		
Omni Max	F´ {*proAB lacIq lacZΔM15* Tn10(TetR) Δ(*ccdAB*)} *mcrA* Δ(*mrr hsdRMS*-*mcrBC*) Φ 80(*lacZ*)ΔM15 Δ(*lacZYA*- *argF*)U169 *endA1 recA1 supE44 thi-1 gyrA96 relA1 tonA panD*	Invitrogen
BL21λDE3	*E*. *coli fhuA2* [lon] *ompT* gal (λ DE3) [dcm] *ΔhsdS* λDE3 (sBamHIo ΔEcoRI-B *int*::(DE3) (*lacI*::PlacUV5::T7 gene1) i21 *Δnin5*)	Invitrogen
ANC108	BL21λDE3/pAC108 (pET21b *6xhis-rsiV*^*+*^)	[38]
LTL408	BL21λDE3/pLL190 (pET21b *6xhis-rsiV*^*A66W*^)	
LTL410	BL21λDE3/pLL192 (pET21b *6xhis-rsiV*^*A66W I76K*^)	
LTL411	BL21λDE3/pLL193 (pET21b *6xhis-rsiV*^*A66W I80K*^)	
*B*. *subtilis* strains		
PY79	Prototrophic derivative of *B*. *subtilis* 168	[59]
CDE1936	Δ*sigVrsiV*::kn *pyrD*::P_*sigV*_-*lacZ*(cat)	
CDE1563	Δ*sigVrsiV*::kn	[16]
LTL172	Δ*sigVrsiV*::kn *amyE*::P_*hs*_-*rsiV*(*spec*) pLL101	
LTL423	Δ*sigVrsiV*::kn *amyE*::P_*hs*_-*rsiV*^*1-66*^*-gfp*(*spec*) pLL197	
LTL424	Δ*sigVrsiV*::kn *amyE*:: P_*hs*_ -*rsiV*^*1-76*^*-gfp*(*spec*) pLL194	
LTL425	Δ*sigVrsiV*::kn *amyE*:: P_*hs*_ -*rsiV*^*1-86*^*-gfp*(*spec*) pLL195	
LTL426	Δ*sigVrsiV*::kn *amyE*:: P_*hs*_ -*rsiV*^*1-96*^*-gfp*(*spec*) pLL196	
LTL144	Δ*sigVrsiV*::kn *pyrD*::P_*sigV*_-*lacZ*(cat) *thrC*::P_*sigV*_-*sigVrsiV*(*erm*) pLL114	
LTL253	Δ*sigVrsiV*::kn *pyrD*::P^*sigV*^-*lacZ*(cat) *thrC*::P_*sigV*_-*sigVrsiV*^*I73K*^(*erm*) pLL201	
LTL254	Δ*sigVrsiV*::kn *pyrD*::P_*sigV*_-*lacZ*(cat) *thrC*::P_*sigV*_-*sigVrsiV*^*I76*K^(*erm*) pLL140	
LTL255	Δ*sigVrsiV*::kn *pyrD*::P_*sigV*_-*lacZ*(cat) *thrC*::P_*sigV*_-*sigVrsiV*^*I80K*^(*erm*) pLL141	
LTL430	Δ*sigVrsiV*::kn *pyrD*::P_*sigV*_-*lacZ*(cat) *thrC*::P_*sigV*_-*sigVrsiV*^*M670K*^(*erm*) pLL198	
LTL456	Δ*sigVrsiV*::kn *pyrD*::P_*sigV*_-*lacZ*(cat) *thrC*::P_*sigV*_-*sigVrsiV*^*Δ67-76*^(*erm*) pLL208	
LTL457	Δ*sigVrsiV*::kn *pyrD*::P_*sigV*_-*lacZ*(cat) *thrC*::P_*sigV*_-*sigVrsiV*^*Δ67-86*^(*erm*) pLL209	
LTL464	Δ*sigVrsiV*::kn *amyE*:: P_*hs*_ -*rsiV*^*Δ67-76*^(*spec*) pLL212	
LTL465	Δ*sigVrsiV*::kn amyE:: P_*hs*_ -*rsiV*^*Δ67-86*^(*spec*) pLL213	
LTL279	Δ*sigVrsiV*::kn amyE:: P_*hs*_ -*6xhis-rsiV*^*A66W*^(*spec*) pLL158	
LTL280	Δ*sigVrsiV*::kn *amyE*:: P_*hs*_ -*6xhis-rsiV*^*Q65C A66W*^(*spec*) pLL159	
LTL433	Δ*sigVrsiV*::kn *amyE*:: P_*hs*_ -*6xhis-rsiV*^*A66W M67C*^(*spec*)pLL185	
LTL437	Δ*sigVrsiV*::kn *amyE*:: P_*hs*_ -*6xhis-rsiV*^*A66W K69C*^(*spec*) pLL202	
LTL356	Δ*sigVrsiV*::kn *amyE*:: P_*hs*_ -*6xhis-rsiV*^*A66W I70C*^(*spec*) pLL182	
LTL438	Δ*sigVrsiV*::kn *amyE*:: P_*hs*_ -*6xhis-rsiV*^*A66W V72C*(^*spec*) pLL203	
LTL318	Δ*sigVrsiV*::kn *amyE*:: P_*hs*_ -*6xhis-rsiV*^*A66W I73C*^(*spec*) pLL165	
LTL319	Δ*sigVrsiV*::kn *amyE*:: P_*hs*_ -*6xhis-rsiV*^*A66W I76C*^(*spec*) pLL167	
LTL348	Δ*sigVrsiV*::kn *amyE*:: P_*hs*_ -*6xhis-rsiV*^*A66W K78C*^(*spec*) pLL179	
LTL357	Δ*sigVrsiV*::kn *amyE*:: P_*hs*_ -*6xhis-rsiV*^*A66W I80C*^(*spec*) pLL193	
LTL358	Δ*sigVrsiV*::kn *amyE*:: P_*hs*_ -*6xhis-rsiV*^*A66W F82C*^(*spec*) pLL184	
LTL316	Δ*sigVrsiV*::kn *amyE*:: P_*hs*_ -*6xhis-rsiV*^*A66W E84C*^(*spec*) pLL168	
LTL315	Δ*sigVrsiV*::kn *amyE*:: P_*hs*_ -*6xhis-rsiV*^*A66W A167C*^(*spec*) pLL169	
LTL474	Δ*sigVrsiV*::kn *pyrD*::P_*sigV*_-*lacZ*(cat) *thrC*::P_*sigV*_-*sigVrsiV*^*Q65C*^(*erm*) pLL215	
LTL475	Δ*sigVrsiV*::kn *pyrD*::P_*sigV*_-*lacZ*(cat) *thrC*::P_*sigV*_-*sigVrsiV*^*M67C*^(*erm*) pLL216	
LTL476	Δ*sigVrsiV*::kn *pyrD*::P_*sigV*_-*lacZ*(cat) *thrC*::P_*sigV*_-*sigVrsiV*^*K69C*^(*erm*) pLL217	
LTL477	Δ*sigVrsiV*::kn *pyrD*::P_*sigV*_-*lacZ*(cat) *thrC*::P_*sigV*_-*sigVrsiV*^*I70C*^(*erm*) pLL218	
LTL478	Δ*sigVrsiV*::kn *pyrD*::P_*sigV*_-*lacZ*(cat) *thrC*::P_*sigV*_-*sigVrsiV*^*V72C*(^*erm*) pLL219	
LTL450	Δ*sigVrsiV*::kn *pyrD*::P_*sigV*_-*lacZ*(cat) *thrC*::P_*sigV*_-*sigVrsiV*^*I73C*^(*erm*) pLL205	
LTL271	Δ*sigVrsiV*::kn *pyrD*::P_*sigV*_-*lacZ*(cat) *thrC*::P_*sigV*_-*sigVrsiV*^*I76C*^(*erm*) pLL153	
LTL510	Δ*sigVrsiV*::kn *pyrD*::P_*sigV*_-*lacZ*(cat) *thrC*::P_*sigV*_-*sigVrsiV*^*K78C*^(*erm*) pLL206	
LTL452	Δ*sigVrsiV*::kn *pyrD*::P_*sigV*_-*lacZ*(cat) *thrC*::P_*sigV*_-*sigVrsiV*^*I80C*^(*erm*) pLL207	
LTL479	Δ*sigVrsiV*::kn *pyrD*::P_*sigV*_-*lacZ*(cat) *thrC*::P_*sigV*_-*sigVrsiV*^*F82C*^(*erm*) pLL220	
LTL273	Δ*sigVrsiV*::kn *pyrD*::P_*sigV*_-*lacZ*(cat) *thrC*::P_*sigV*_-*sigVrsiV*^*E84C*^(*erm*) pLL155	
LTL503	Δ*sigVrsiV*::kn *amyE*:: P_*hs*_ -*6xHis-rsiV*^*A66W V72C I80K*^(*spec*) pLL222	
LTL504	Δ*sigVrsiV*::kn *amyE*:: P_*hs*_ -*6xHis-rsiV*^*A66W I76C I80K*^(*spec*) pLL223	

**Table 3:** Plasmid list.

Plasmid	Features	Reference
pDR111	*amyE* P_*hs*_ Amp^r^ Spec^r^	[68]
pDG1664	*thrC* Amp^r^ Erm^r^ Spec^r^	[69]
pCM11	sGFP	[62]
pET21b	Amp^r^, N terminal-6xhis expression vector, T7 promoter	
pCE544	pDR111 *6xhis-rsiV*	
pLL190	pET21b *rsiV*^*A66W*^	
pLL192	pET21b *rsiV*^*A66W I76K*^	
pLL193	pET21b *rsiV*^*A66W I80K*^	
pLL101	pDR111 *amyE*::P_*hs*_-*rsiV*(*spec*)	
pLL197	pDR111 *amyE*::P_*hs*_-^*rsiV1-66*^*-gfp*(*spec*)	
pLL194	pDR111 *amyE*::P_*hs*_-*rsiV*^*1-76*^*-gfp*(*spec*)	
pLL195	pDR111 *amyE*:: P_*hs*_-*rsiV*^*1-86*^*-gfp*(*spec*)	
pLL196	pDR111 *amyE*:: P_*hs*_-*rsiV*^*1-96*^*-gfp*(*spec*)	
pLL114	pDG1664 *thrC*::P_*sigV*_-*sigVrsiV*(erm)	
pLL201	pDG1664 *thrC*:: P_*sigV*_ -*sigVrsiV*^*I73K*^(*erm*)	
pLL140	pDG1664 *thrC*:: P_*sigV*_ -*sigVrsiV*^*I76K*^(*erm*)	
pLL141	pDG1664 *thrC*:: P_*sigV*_ -*sigVrsiV*^*I80K*^(*erm*)	
pLL198	pDG1664 *thrC*:: P_*sigV*_ -*sigVrsiV*^*M67K*^(*erm*)	
pLL208	pDG1664 *thrC*:: P_*sigV*_ -*sigVrsiV*^*Δ67*-*76*^(*erm*)	
pLL209	pDG1664 *thrC*:: P_*sigV*_ -*sigVrsiV*^*Δ67*-*86*^(*erm*)	
pLL212	pDR111 *amyE*:: P_*hs*_ -*rsiV*^*Δ67-76*^(*spec*)	
pLL213	pDR111 *amyE*:: P_*hs*_ -*rsiV*^*Δ67-86*^*(spec*)	
pLL158	pDR111 *amyE*:: P_*hs*_ -*6xhis-rsiV*^*A66W*^(*spec*)	
pLL159	pDR111 *amyE*:: P_*hs*_ -*6xhis-rsiV*^*Q65C A66W*^(*spec*)	
pLL185	pDR111 *amyE*:: P_*hs*_ -*6xhis-rsiV*^*A66W M67C*^(*spec*)	
pLL202	pDR111 *amyE*:: P_*hs*_ -*6xhis-rsiV*^*A66W K69C*^(*spec*)	
pLL182	pDR111 *amyE*:: P_*hs*_ -*6xhis-rsiV*^*A66W I70C*^(*spec*)	
pLL203	pDR111 *amyE*:: P_*hs*_ -*6xhis-rsiV*^*A66W V72C*^(*spec*)	
pLL165	pDR111 *amyE*:: P_*hs*_ -*6xhis-rsiV*^*A66W I73C*^(*spec*)	
pLL167	pDR111 *amyE*:: P_*hs*_ -*6xhis-rsiV*^*A66W I76C*^(*spec*)	
pLL179	pDR111 *amyE*:: P_*hs*_ -*6xhis-rsiV*^*A66W K78C*^(*spec*)	
pLL193	pDR111 *amyE*:: P_*hs*_ -*6xhis-rsiV*^*A66W I80C*^(*spec*)	
pLL184	pDR111 *amyE*:: P_*hs*_ -*6xhis-rsiV*^*A66W F82C*^(*spec*)	
pLL168	pDR111 *amyE*:: P_*hs*_ -*6xhis-rsiV*^*A66W E84C*^(*spec*)	
pLL169	pDR111 *amyE*:: P_*hs*_ -*6xhis-rsiV*^*A66W A167C*^(*spec*)	
pLL215	pDG1664 *thrC*:: P_*sigV*_ -*sigVrsiV*^*Q65C*^(*erm*)	
pLL216	pDG1664 *thrC*:: P_*sigV*_ -*sigVrsiV*^*M67C*^(*erm*)	
pLL217	pDG1664 *thrC*:: P_*sigV*_ -*sigVrsiV*^*K69C*^(*erm*)	
pLL218	pDG1664 *thrC*:: P_*sigV*_ -*sigVrsiV*^*I70C*(^*erm*)	
pLL219	pDG1664 *thrC*:: P_*sigV*_ -*sigVrsiV*^*V72C*^(*erm*)	
pLL205	pDG1664 *thrC*:: P_*sigV*_ -*sigVrsiV*^*I73C*^(*erm*)	
pLL153	pDG1664 *thrC*:: P_*sigV*_ -*sigVrsiV*^*I76C*^(*erm*)	
pLL206	pDG1664 *thrC*:: P_*sigV*_ -*sigVrsiV*^*K78C*^(*erm*)	
pLL207	pDG1664 *thrC*:: P_*sigV*_ -*sigVrsiV*^*I80C*^(*erm*)	
pLL220	pDG1664 *thrC*:: P_*sigV*_ -*sigVrsiV*^*F82C*^(*erm*)	
pLL155	pDG1664 *thrC*:: P_*sigV*_ -*sigVrsiV*^*E84C*^(*erm*)	
pLL222	pDR111 *amyE*::P_*hs*_-*6xHis-rsiV*^*A66W V72C I80K*^(*spec*)	
pLL223	pDR111 *amyE*::P_*hs*_-*6xHis-rsiV*^*A66W I76C I80K*^(*spec*)	

Constructs were cloned into pDR111 in order to place *rsiV* variants under control of the IPTG-inducible hyper-spank (P_*hs*_) promoter. The vector, pDR111 was digested with HindIII and SphI and PCR products were introduced by isothermal assembly. All pDR111 constructs were transformed into CDE1563 (*ΔsigVrsiV*::*kn*) and the RsiV construct was introduced at the *amyE* gene on the chromosome. WT RsiV was created using CDEP3194/CDEP3195 (chromosomal DNA). Rsiv-GFP fusion construct inserts were created using CDEP3865/Appropriate RsiV-GFP Reverse (Rev) primer (chromosomal DNA) and CDEP3868/Appropriate RsiV-GFP Forward (For) primer (pCM11 template) [62]. RsiV deletion constructs were created using CDEP3194/Appropriate RsiVΔ Rev primer (chromosomal DNA) and CDEP3195/Appropriate RsiVΔ For primer. SCAM constructs were created by first building 6xHis-rsiV^*A66W*^ using primers CDEP3629/A66W Rev (chromosomal DNA) and CDEP3195/A66W For (chromosomal DNA). The remaining SCAM constructs were created using 6xHis-rsiV^*A66W*^ as template. The inserts were created with primers CDEP3629/Appropriate *rsiV* For primer and CDEP3195/Appropriate *rsiV* Rev.

Constructs cloned onto pDG1664 were created by first digesting the vector with BamHI and EcoRI to allow for Isothermal Assembly of the PCR products. All pDG1664 constructs were transformed into CDE1936 (Δ*sigVrsiV*::*kn pyrD*::P_*sigV*_-*lacZ*(*cat*)) and the *sigVrsiV* constructs were introduced at the *thrC* gene on the chromosome. WT *sigVrsiV* was created using primers CDEP3533/CDEP3534 (*sigVrsiV* template). Deletions constructs, lysine substitutions, and cysteine mutations in the reporter strain were created with the primers CDEP3533/Appropriate *rsiV* Rev and CDEP3534/Appropriate *rsiV* For (*sigVrsiV* template).

Constructs cloned onto pET21b were created by first digesting the vector with BamHI and NdeI to allow for Isothermal Assembly of the PCR products. All pET21b constructs were transformed into *E*. *coli* BL21λDE3. ANC108 (pET21b *6xhis-rsiV*)[38] was used as the initial template to create the constructs. First, we generated *6xhis-rsiV*^*A66W*^ using the primers CDEP3165/1562 and CDEP3267/CDEP1561. We then used *6xhis-rsiV*^*A66W*^ as template to create *6xhis-rsiV*^*A66W I76K*^ and *6xhis-rsiV*^*A66W I80K*^ using the primers CDEP3165/Appropriate *rsiV* Rev and CDEP3267/Appropriate *rsiV* For.

### Media supplements

Antibiotics were used at the following concentrations: chloramphenicol, 5 μg/ml; MLS erythromycin plus lincomycin, 1 μg/ml and 25 μg/ml; kanamycin, 5 μg/ml; spectinomycin, 100 μg/ml; ampicillin 100 μg/ml. Isopropyl β-D-1-thiogalactopyranoside (IPTG) was used at a final concentration of 1 mM unless otherwise noted.

### Protein expression and concentration

Cell cultures were grown overnight in LB broth at 37°C and subcultured 1:100 in 5 mL LB IPTG (1mM). Cells were incubated at 37°C for ~3hrs to an OD_600_ of 0.8–1 and 1 mL of cell culture was centrifuged to obtain a pellet and supernatant. The proteins in the supernatant were precipitated with methanol chloroform [63] and the resulting precipitant was analyzed by western blotting. The culture pellet was resuspended in 100 μL LB with or without lysozyme (10 μg/mL) and incubated at room temperature. After 10 min, 100 μL 2x Laemmli sample buffer was added to stop the reaction. Cells were sonicated with a Branson Sonifier 450 and analyzed by western blotting.

### β-galactosidase assay

Cells were grown in LB (1% NaCl) to inhibit lysozyme activity [64]. ON cells were subcultured 1:20 and grown at 37°C for 2 hrs. These early log-phase cultures were then diluted to an OD_600_ of 0.05 and grown in 200 μL volumes in a round bottom 96-well plate in the presence of varied concentrations of lysozyme (0, 5, and 10 μg/mL). The plate was shaken at 1000 rpm at 37°C for 4 hrs. The cell density (OD_600_) was determined by diluting 50 μL from each well into 150 μL LB in a 96-well flat bottom plate. The remainder of each culture was lysed by mixing with 6 μL 2% sarkosyl, 12 μL chloroform. Lysate (20 μL) was added to 100 μL Z-buffer (60mM Na_2_HPO_4_, 40mM NaH_2_PO_4_, 10mM KCl, 1mM MgSO_4_, 50mM 2-mercaptoethanol, pH 7.0) in a flat bottom 96-well plate. The reaction was started by the addition of 50 μL ONPG (10 μg/mL) and color development followed at 405 nm, every 1 min for 30 min (Tecan Infinite M200 Pro). β-galactosidase activity was reported as the rate of product development normalized to cell density.

### SCAM assay

This protocol was adapted from [65]. Cells cultures were grown overnight in LB broth at 37°C and subcultured 1:100 in 50 mL LB IPTG. Cells were incubated at 37°C for ~3hrs to an OD_600_ of 0.8–1 and 15 mL cell cultures were centrifuged and pellets resuspended in 500 μL protoplast buffer (0.4 M sucrose, 10 mM KPi, 15 mM MgCl_2_) [66] with and without lysozyme (75 μg/mL) and incubated at room temperature. After 10 minutes Na-(3-maleimidylpropionyl) biocytin (MPB, Invitrogen) (50 μM) was added to label cysteine residues. The reaction was quenched by the addition of 2-mercaptoethanol to a final concentration 20 mM. Cells were washed with protoplast buffer amended with 2-mercaptoethanol (20 mM).

Labeled RsiV variants were then affinity purified as described below, with volumes adjusted to the smaller scale: 50 μL nickel resin, 500 μL lysis buffer, 500 μL wash buffer, and 50 μL elution buffer. The eluted protein was immediately mixed with 50 μL 2X Laemmli buffer and analyzed by immunoblotting.

### Immunoblot analysis

Samples were electrophoresed on a 15% SDS polyacrylamide gel (Biorad) and proteins were then blotted onto a nitrocellulose membrane (GE Healthcare, Amersham). Nitrocellulose was blocked with 5% Bovine Serum Albumin (BSA) and proteins were detected with either 1:10,000 dilution anti-RsiV^59-285^ [16] or anti-GFP. Streptavidin IR680LT (1:2500) was used to detect two biotin-containing proteins, PycA and AccB, which serve as loading controls [67]. To detect primary antibodies, nitrocellulose was washed and incubated with 1:10,000 dilution of Goat anti-Rabbit IR800CW (Li-Cor) and imaged on an Odyssey CLx Scanner (Li-Cor). All immunoblots were performed a minimum of three times with a representative example shown.

### Protein purification

Expression cultures of RsiV variants were prepared as previously described [37]. Briefly, 50 mL of LB Amp100 was inoculated 1:100 with overnight cultures of BL21(DE3) harboring the respective expression plasmids, grown shaking at 37°C to an OD_600_ of 0.8, induced with 1 mM IPTG and shifted to 30°C. After 3 hours of growth, cells were pelleted by centrifugation. RsiV variants included a 6xHis-tag at the N-terminus and were affinity purified over Nickel resin (Thermo HisPur Ni-NTA, Cat Nr 88222). Batch purification was performed in 50 mM TrisHCl, pH 8.0, 250 mM NaCl, all steps at 4°C. Lysis buffer, wash buffer and elution buffer included 10 mM, 20 mM and 250 mM imidazole, respectively. Nickel resin (500 μL slurry) was equilibrated with lysis buffer. Cell pellets were lysed in 2 mL lysis buffer by sonication (Branson Sonifier 450, microtip, output 3, three cycles of 30 pulses) and the lysate was clarified by spinning at 30 min 16,000 x g. The supernatant was incubated with the prepared resin for 20 min, washed five times with 2 mL wash buffer and protein was eluted in two 1 mL fractions. The fractions were combined and dialyzed against 50 mM NaPi, pH 7.0, 100 mM NaCl.

### Circular dichroism

CD spectroscopy to determine protein secondary structure was performed with about 10 μM protein in 50 mM NaPi, pH 7.0, 100 mM NaCl. CD spectra were recorded from 260 nm to 190 nm with 1 nm data interval at 25°C in a Jasco J-815 CD spectropolarimeter. Data integration time was 2 seconds and the scanning speed was 100 nm/min. For comparison to WT protein, spectra were normalized to the signal at 220 nm.

## Supporting information

S1 Fig Sequence logo of RsiV amphipathic helices. An alignment of 203 RsiV homologues was used to create a sequence logo mapping frequency of residues that correspond to *B*. *subtilis* RsiV 57–85. Hydrophobic residues are in blue and hydrophilic residues are in red. The cleavage site is marked by a red arrow.
(TIF)

S2 Fig Amino acids after the cleavage site are involved in the protection of RsiV-GFP from signal peptidase cleavage. Cells producing various lengths of N-terminal RsiV (1–66, 1–76, 1–86 and 1–96) fused to GFP were grown to mid log. Cell pellets and the supernatants were collected and analyzed by western blot with α-RsiV^59-258^ antibodies. Streptavidin IR680LT was used detect PycA which served as a loading control [67].
(TIF)

S3 Fig Deletions of RsiV after the cleavage site lead to constitutive RsiV degradation. Deletions in RsiV after the cleavage site were created (RsiV^Δ67–76^, RsiV^Δ67–86^) and expressed under an IPTG inducible promoter. Cell pellets and the supernatants were collected, and the pellets were exposed to -/+ lysozyme (10μg/mL). Samples were analyzed by western blot with α-RsiV^59-258^ antibodies and streptavidin IR680LT was used detect PycA which served as a loading control [67].
(TIF)

S4 Fig Deletion of residues after cleavage site lead to constitutive cleavage. The constructs RsiV^Δ67–76^ and RsiV^Δ67–86^ used to measure σ^V^ activity were further analyzed by western blot to measure RsiV degradation. Cells were grown to mid-log. The pellet and supernatants were collected, and samples were analyzed by western blot with α-RsiV^59-258^ antibodies. Streptavidin IR680LT was used detect PycA which served as a loading control [67].
(TIF)

S5 Fig Lysine substitutions in the amphipathic helices lead to constitutive RsiV cleavage. The lysine substitution constructs (M67K, I73K, I76K, I80K) were analyzed by western blot to measure RsiV degradation. Cells were grown to mid log. The pellet and supernatants were collected, and samples were analyzed by western blot with α-RsiV^59-258^ antibodies. Streptavidin IR680LT was used detect PycA which served as a loading control [67].
(TIF)

S6 Fig Lysine substitutions do not affect secondary structure. Lysine substitution constructs (I76C and I80C) were combined with an N-term 6xHis tag and an A66W substitution that blocks signal peptidase activity to allow for purification of the constructs. WT RsiV and A66W were also purified to serve as controls for the experiment. CD analysis was performed and normalized to 220nm to determine if the lysine substitution caused abnormalities in the secondary structure.
(TIF)

S7 Fig Cysteine substitutions do not affect RsiV cleavage. The cysteine substitutions were analyzed by western blot to measure RsiV degradation. Cells were grown to mid log. The pellet and supernatants were collected, and samples were analyzed by western blot with α-RsiV^59-285^ antibodies. Streptavidin IR680LT was used detect PycA which served as a loading control [67].
(TIF)

S1 Table Oligonucleotides. (PDF)
