# Supplementary figures and images for: Bacterial sensing: A putative amphipathic helix in RsiV is the switch for activating σV in response to lysozyme
Source: PLoS Genet. 2018 Jul 18;14(7):e1007527. doi: 10.1371/journal.pgen.1007527 (PMC6066255; doi:10.1371/journal.pgen.1007527)

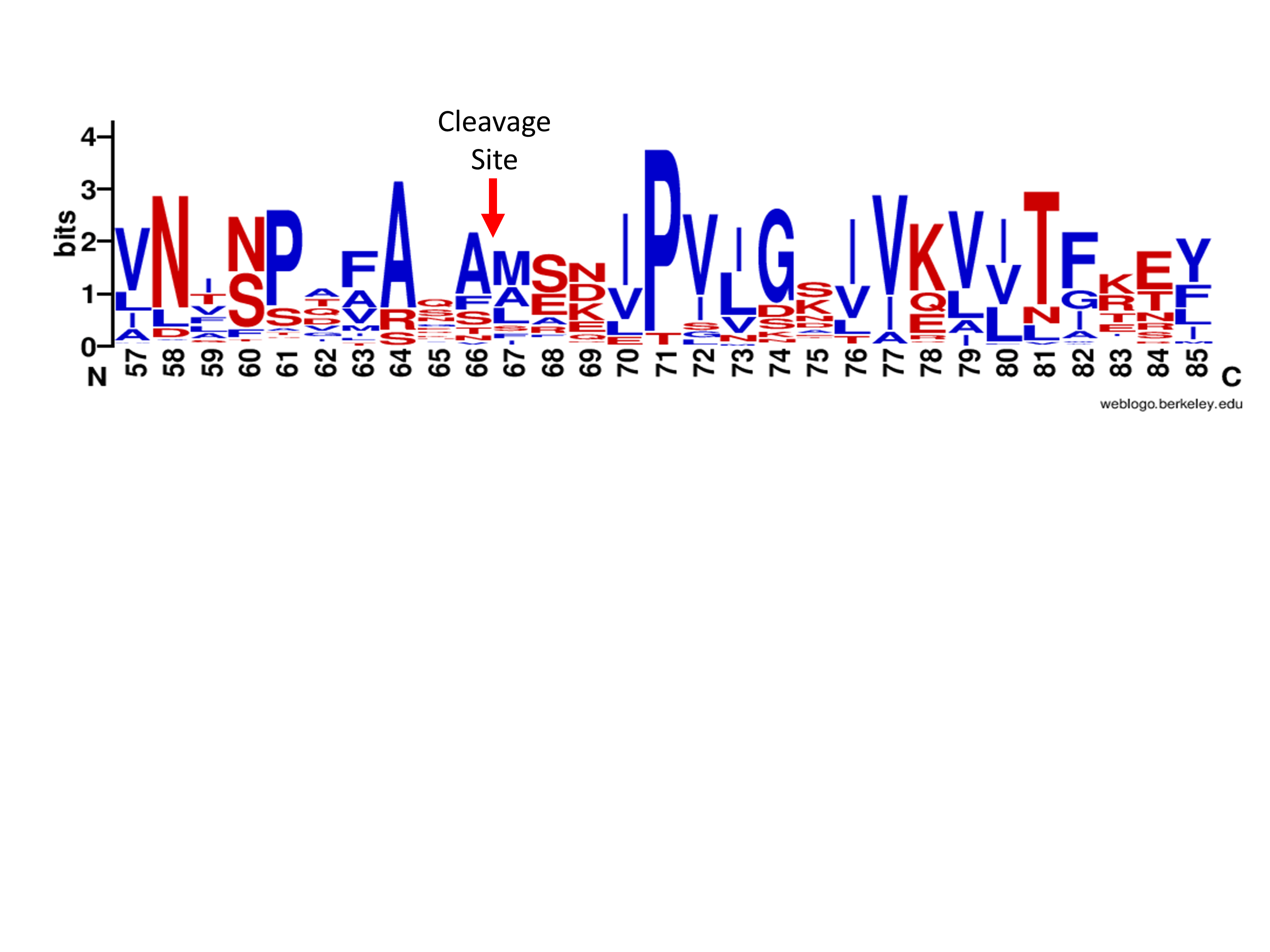

Supplement: S1 Fig — An alignment of 203 RsiV homologues was used to create a sequence logo mapping frequency of residues that correspond to B. subtilis RsiV 57–85. Hydrophobic residues are in blue and hydrophilic residues are in red. The cleavage site is marked by a red arrow. (TIF) [file pgen.1007527.s001.TIF]

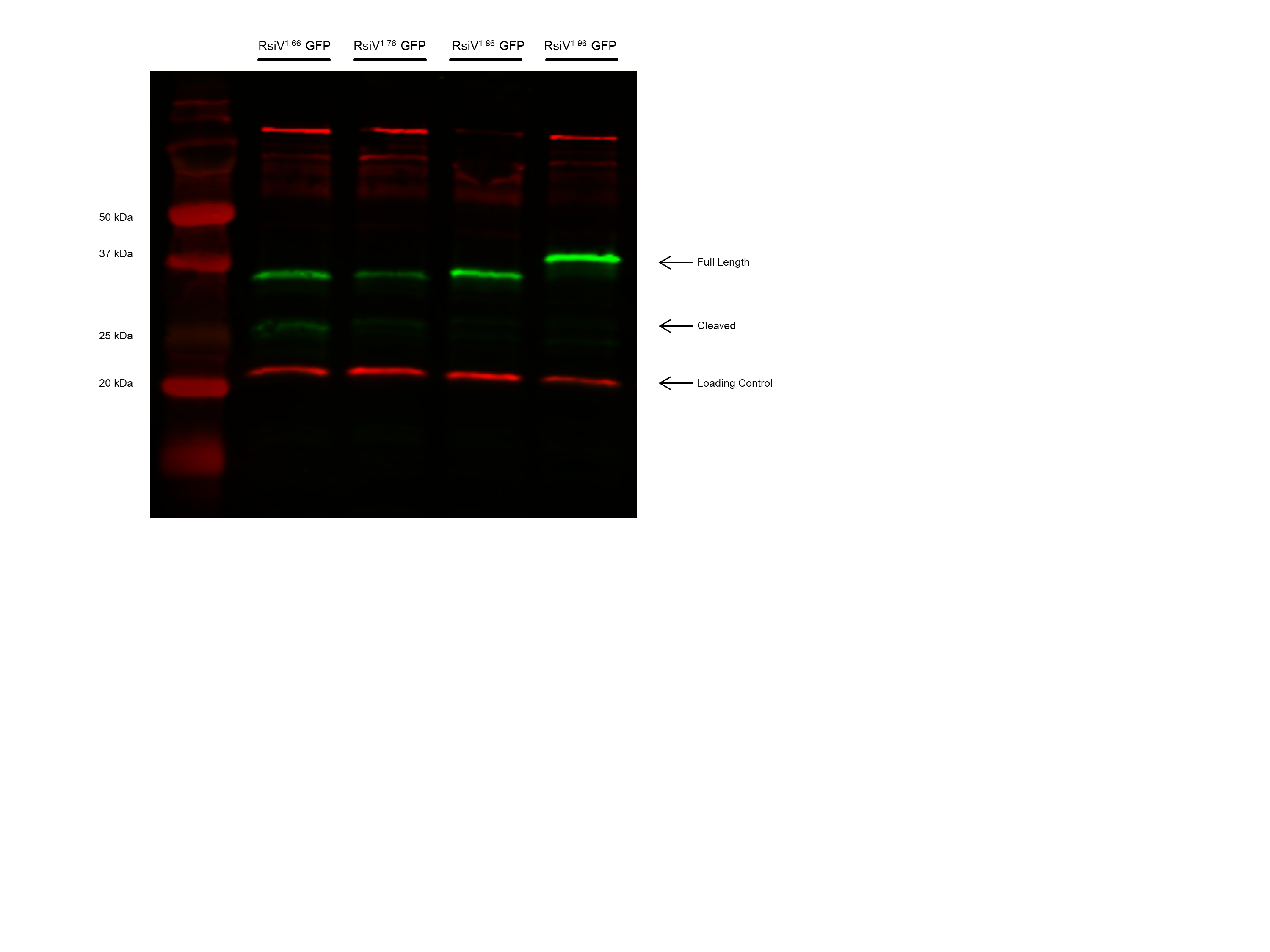

Supplement: S2 Fig — Cells producing various lengths of N-terminal RsiV (1–66, 1–76, 1–86 and 1–96) fused to GFP were grown to mid log. Cell pellets and the supernatants were collected and analyzed by western blot with α-RsiV59-258 antibodies. Streptavidin IR680LT was used detect PycA which served as a loading control [67]. (TIF) [file pgen.1007527.s002.TIF]

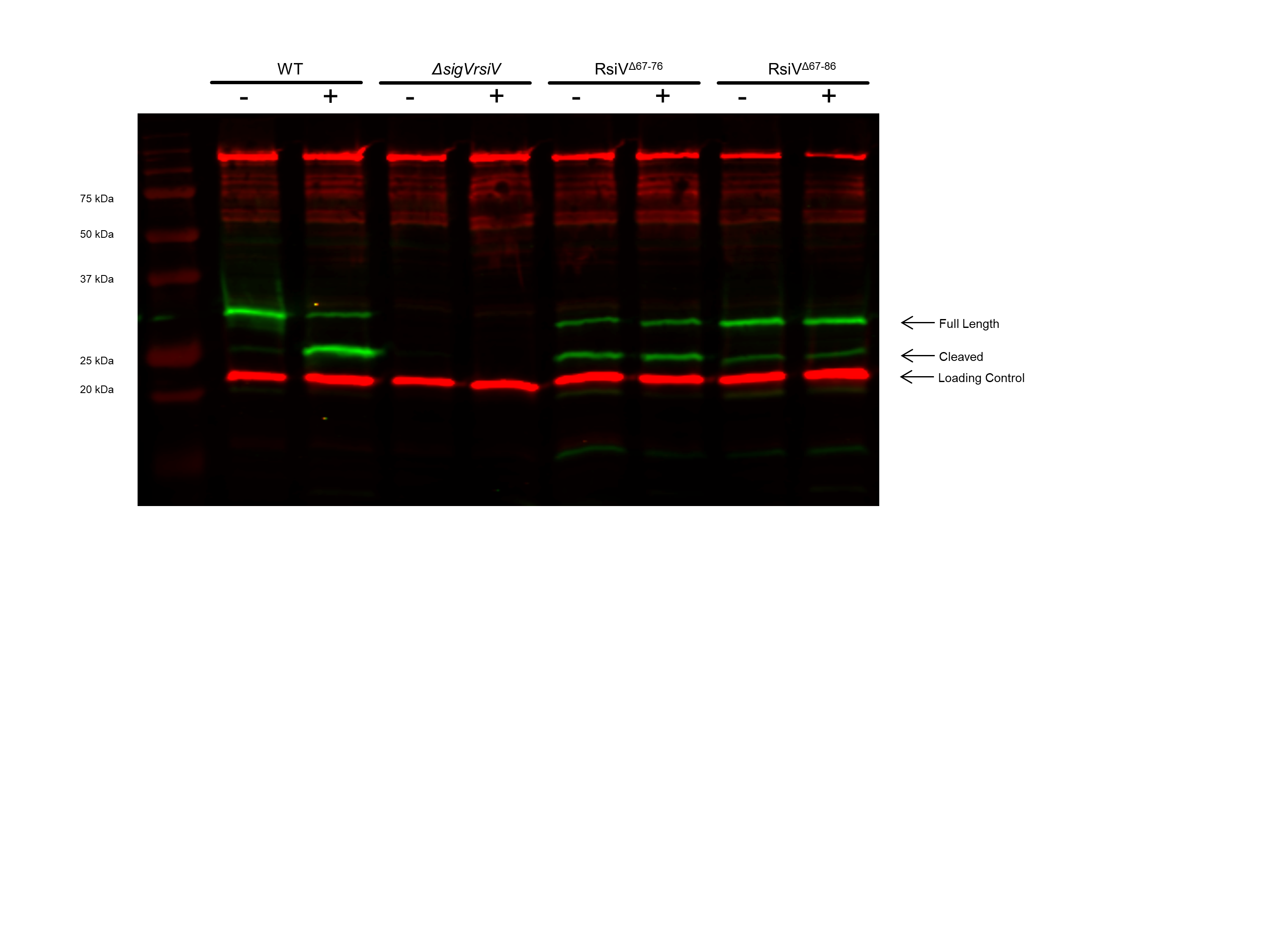

Supplement: S3 Fig — Deletions in RsiV after the cleavage site were created (RsiVΔ67–76, RsiVΔ67–86) and expressed under an IPTG inducible promoter. Cell pellets and the supernatants were collected, and the pellets were exposed to -/+ lysozyme (10μg/mL). Samples were analyzed by western blot with α-RsiV59-258 antibodies and streptavidin IR680LT was used detect PycA which served as a loading control [67]. (TIF) [file pgen.1007527.s003.TIF]

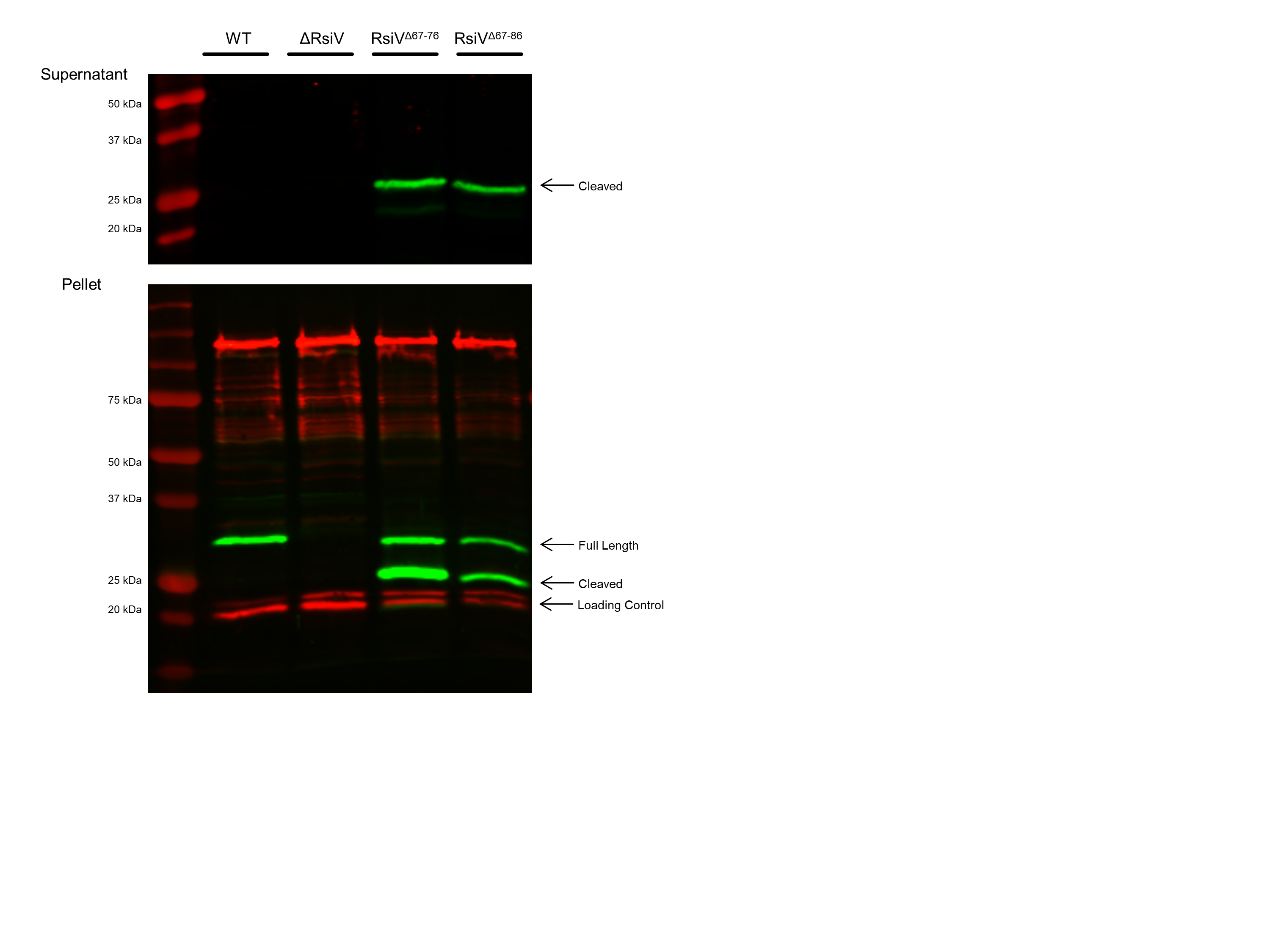

Supplement: S4 Fig — The constructs RsiVΔ67–76 and RsiVΔ67–86 used to measure σV activity were further analyzed by western blot to measure RsiV degradation. Cells were grown to mid-log. The pellet and supernatants were collected, and samples were analyzed by western blot with α-RsiV59-258 antibodies. Streptavidin IR680LT was used detect PycA which served as a loading control [67]. (TIF) [file pgen.1007527.s004.TIF]

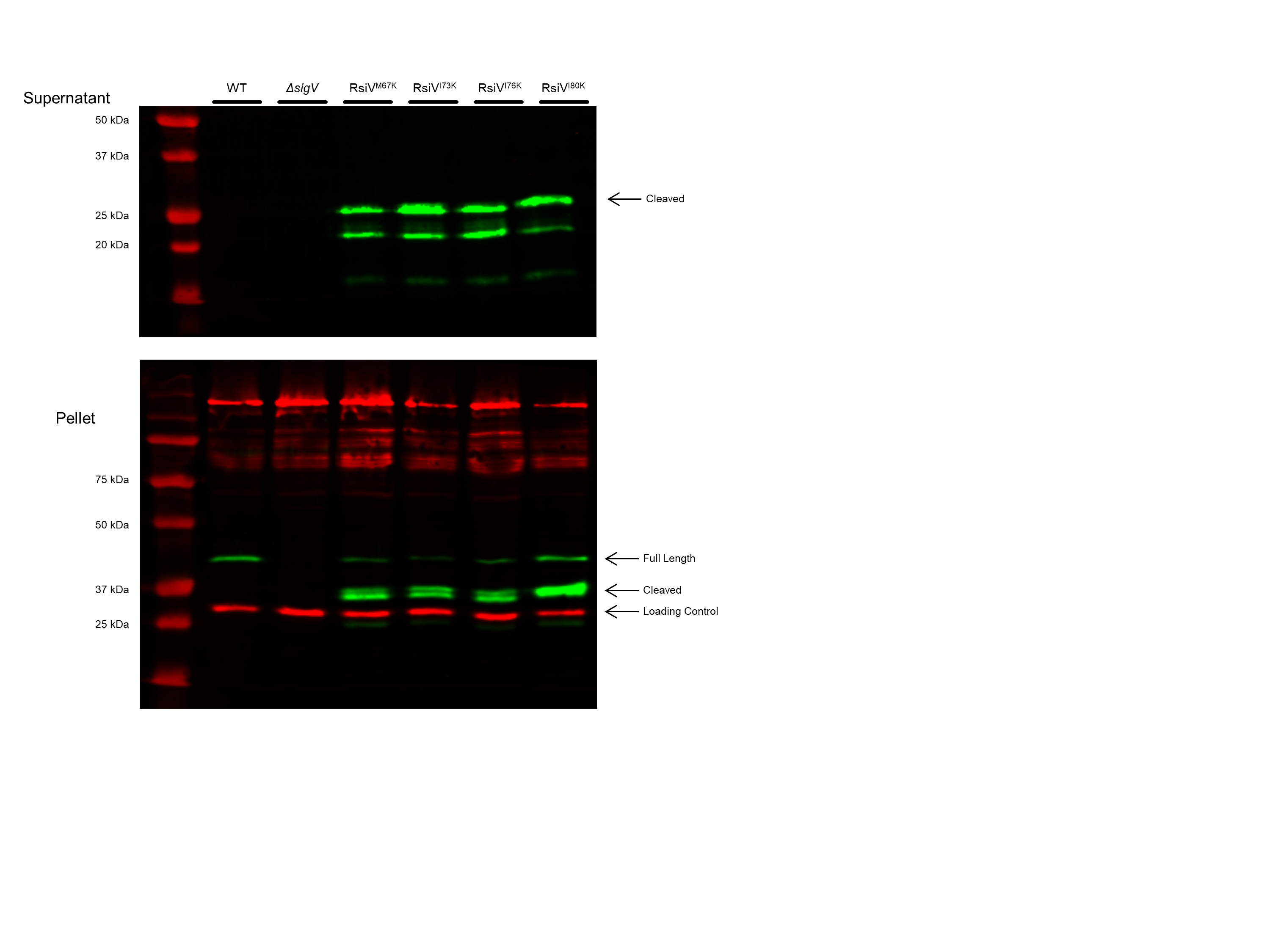

Supplement: S5 Fig — The lysine substitution constructs (M67K, I73K, I76K, I80K) were analyzed by western blot to measure RsiV degradation. Cells were grown to mid log. The pellet and supernatants were collected, and samples were analyzed by western blot with α-RsiV59-258 antibodies. Streptavidin IR680LT was used detect PycA which served as a loading control [67]. (TIF) [file pgen.1007527.s005.TIF]

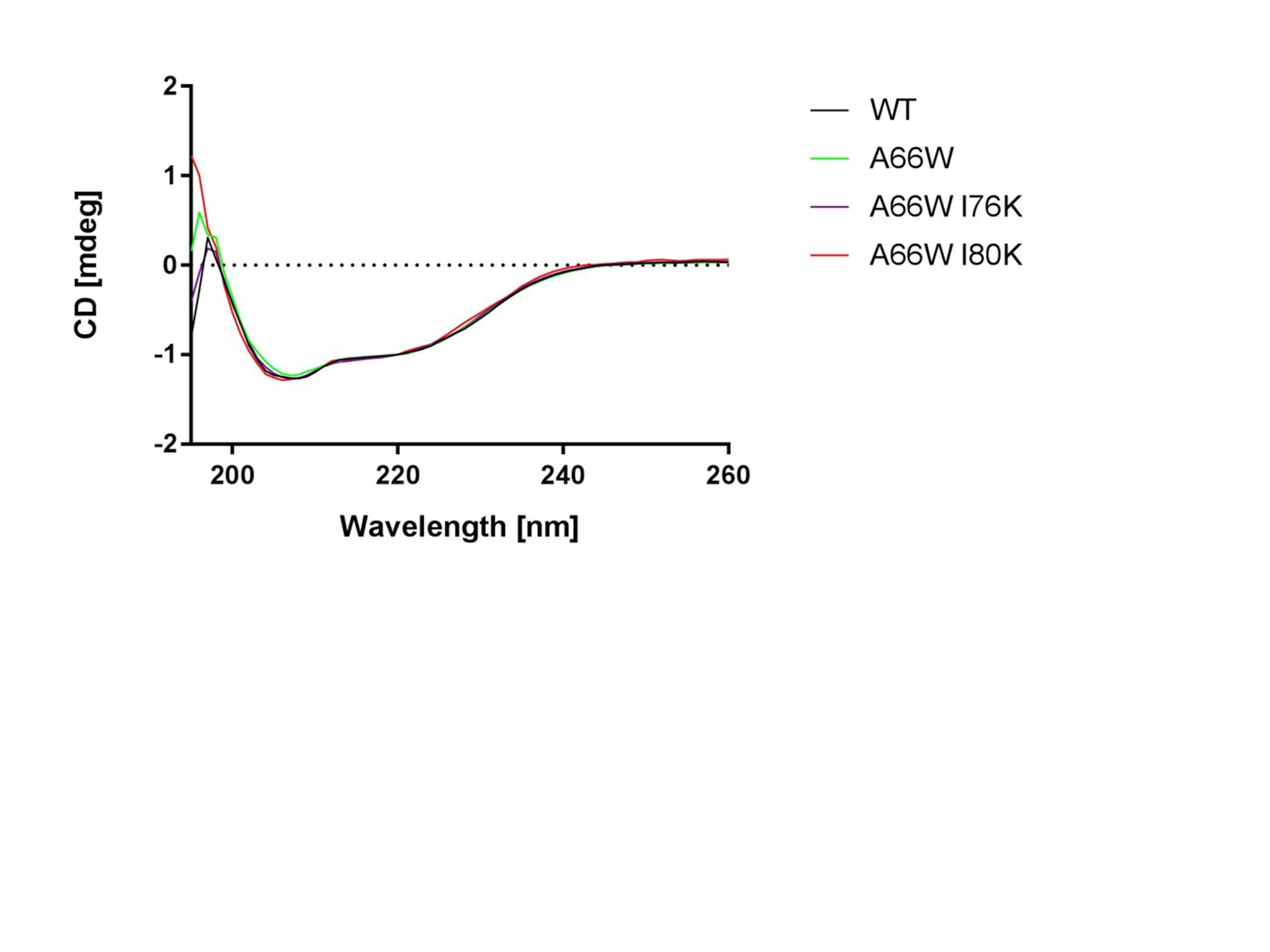

Supplement: S6 Fig — Lysine substitution constructs (I76C and I80C) were combined with an N-term 6xHis tag and an A66W substitution that blocks signal peptidase activity to allow for purification of the constructs. WT RsiV and A66W were also purified to serve as controls for the experiment. CD analysis was performed and normalized to 220nm to determine if the lysine substitution caused abnormalities in the secondary structure. (TIF) [file pgen.1007527.s006.TIF]

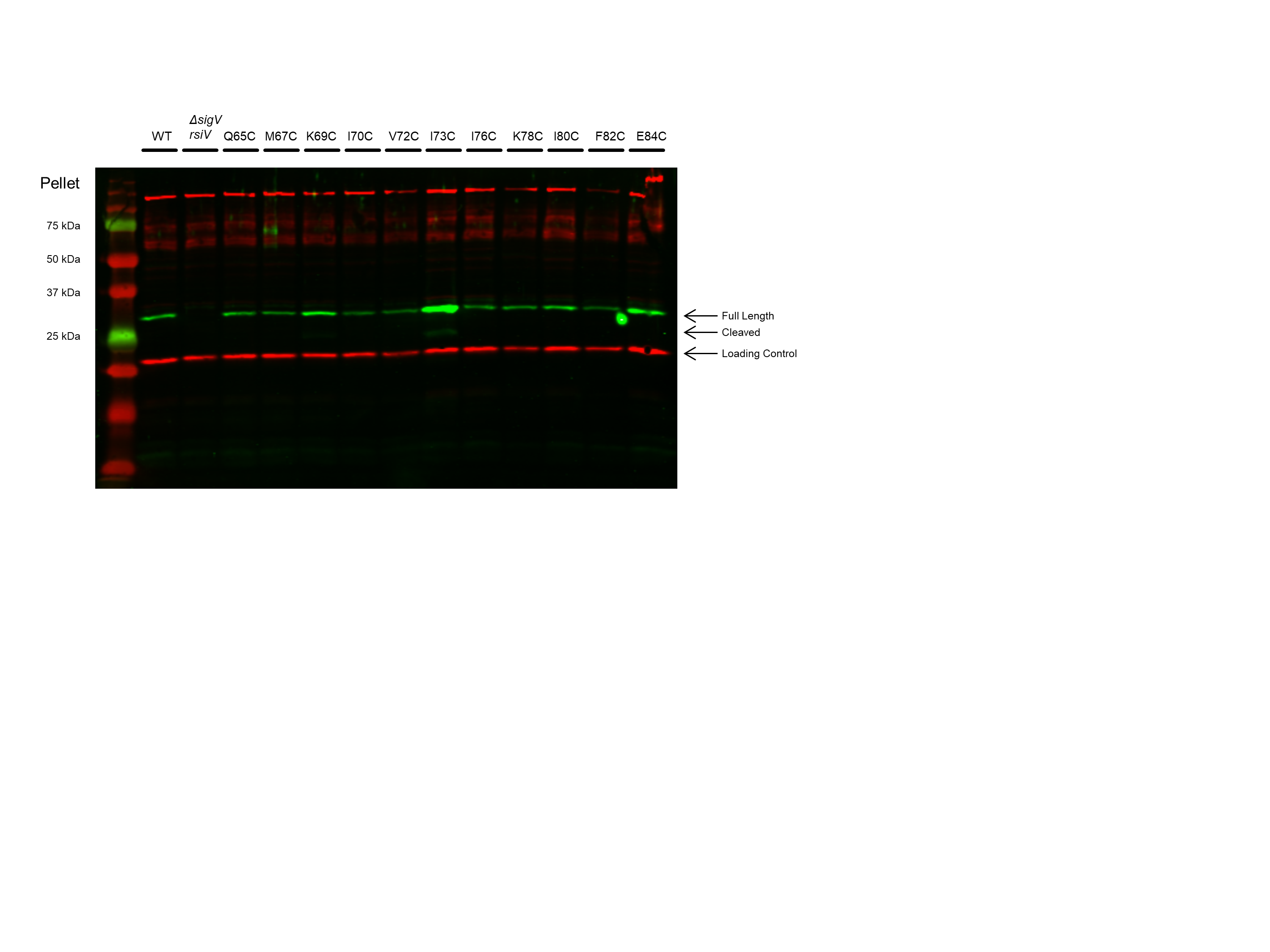

Supplement: S7 Fig — The cysteine substitutions were analyzed by western blot to measure RsiV degradation. Cells were grown to mid log. The pellet and supernatants were collected, and samples were analyzed by western blot with α-RsiV59-285 antibodies. Streptavidin IR680LT was used detect PycA which served as a loading control [67]. (TIF) [file pgen.1007527.s007.TIF]
