# Supplementary material for: Bacterial sensing: A putative amphipathic helix in RsiV is the switch for activating σV in response to lysozyme
Source: PLoS Genet. 2018 Jul 18;14(7):e1007527. doi: 10.1371/journal.pgen.1007527 (PMC6066255; doi:10.1371/journal.pgen.1007527)
Supplement: S1 Table — (PDF) [file pgen.1007527.s008.pdf]

Table S1: Oligonucleotides

Primer	Sequence 5' to 3'	Description of use
CDEP3533	aaaactgctgccttcggatcgggaaaaatctcatccagttg	sigVrsiV pDG1664 For
CDEP3534	aactttagggttatcgaattttagatggatgtttttattttatcgaa	sigVrsiV pDG1664 Rev
CDEP3194	gtgagcggataacaattaagcttaaggaggctttcttaatggataagagattacagcaa	RsiV pDR For
CDEP3865	aacaattaagcttaaggaggctttcttaatggataagagattacagc	RsiV pDR For
CDEP3195	ccgaattagcttgcatgctattttatcgaatataacgttctcc	RsiV pDR Rev
CDEP3868	ccaccgaattagcttgcatgggatcctttgtagagctcat	GFP Outside Rev
CDEP3165	cggagctcgaattcggatccattttatcgaatataacgttctccc	N term 6xHis pET21 For
CDEP3267	tttaagaaggagatatacatatgcaccatcaccatcaccatatggataagagattacagc	RsiV pET21 Rev
CDEP3629	gtgagcggataacaattaagcttaaggaggctttcttaatgcaccatcaccatcaccat	6xHis RsiV pDR For
rsiV 1-66-GFP For	ccggacgccgctcaggctatgagcaaaggagaagaact	pLL197
rsiV 1-76-GFP For	cctgtcatcggcaaaatcatgagcaaaggagaagaact	pLL194
rsiV 1-86-GFP For	acctttattgaaatcaaaatgagcaaaggagaagaact	pLL195
rsiV 1-96-GFP For	caatcaagcattgatgtcatgagcaaaggagaagaact	pLL196
rsiV 1-66-GFP Rev	agttcttctcctttgctcatagcctgagcggcgtccgg	pLL197
rsiV 1-76-GFP Rev	agttcttctcctttgctcatgattttgccgatgacagg	pLL194
rsiV 1-86-GFP Rev	agttcttctcctttgctcattttgatttcaataaaggt	pLL195
rsiV 1-96-GFP Rev	agttcttctcctttgctcatgacatcaatgcttgattg	pLL196
rsiV Δ67-76 For	ccggacgccgctcaggctgtcaaagcgatcaccttt	pLL208, pLL212
rsiV Δ67-86 For	ccggacgccgctcaggctgaggaaaaagaccaatca	pLL209, pLL213
rsiV Δ67-76 Rev	aaaggtgatcgctttgacagcctgagcggcgtccgg	pLL208, pLL212
rsiV Δ67-86 Rev	tgattggtctttttcctcagcctgagcggcgtccgg	pLL209, pLL213
I73K For	atgtcaaagatccctgtcaagggcaaaatcgtcaaagcg	pLL201
I76K For	atccctgtcatcggcaaaaaggtcaaagcgatcaccttt	pLL140
I80K For	ggcaaaatcgtcaaagcgaagacctttattgaaatcaaa	pLL141
M67K For	ccggacgccgctcaggctaagtcaaagatccctgtcatc	pLL198
I73K Rev	cgctttgacgattttgcccttgacagggatctttgacat	pLL201
I76K Rev	aaaggtgatcgctttgacctttttgccgatgacagggat	pLL140
I80K Rev	tttgatttcaataaaggtcttcgctttgacgattttgcc	pLL141
M67K Rev	gatgacagggatctttgacttagcctgagcggcgtccgg	pLL198
A66W For	atcaacccggacgcc gct cag tgg atgtcaaagatccct	pLL158
Q65C A66W For	aatatcaacccggacgccgcttgc gct atgtcaaagatccc	pLL159
A66W M67C For	ccggacgccgctcagtggtgttcaaagatccctgtcatc	pLL185
A66W K69C For	gctcagtggatgtcatgtatccctgtcatcggcaaa	pLL202
A66W I70C For	gctcagtggatgtcaaagtgtcctgtcatcggcaaaatc	pLL182
A66W V72C For	tggatgtcaaagatcccttgtatcggcaaaatcgtcaaa	pLL203
I73C For	atgtcaaagatccctgtctgcggcaaaatcgtcaaagcg	pLL165, pLL205
I76C For	atccctgtcatcggcaaatgcgtcaaagcgatcaccttt	pLL167, pLL153
K78C For	gtcatcggcaaaatcgtctgtgcgatcacctttattgaa	pLL179, pLL206
I80C For	ggcaaaatcgtcaaagcgtgtacctttattgaaatcaaa	pLL193, pLL207
F82C For	atcgtcaaagcgatcacctgtattgaaatcaaagaggaa	pLL184, pLL220
E84C For	aaagcgatcacctttatttgcatcaaagaggaaaaagac	pLL168, pLL155
A167C For	ccggcgcaatattgaaacaacacaatgctcttcctacacacaaagccg	pLL169
A66W Rev	agggatctttgacatccactgagcggcgtccgggttgat	pLL158
Q65C A66W Rev	gggatctttgacatagcgcaagcggcgtccgggttgatatt	pLL159
A66W M67C Rev	gatgacagggatctttgaacaccactgagcggcgtccgg	pLL185
A66W K69C Rev	tttgccgatgacagggatacatgacatccactgagc	pLL202
A66W I70C Rev	gattttgccgatgacaggacactttgacatccactgagc	pLL182

A66W V72C Rev	tttgacgattttgccgatacaagggatctttgacatcca	pLL203
I73C Rev	cgctttgacgattttgccgcagacagggatctttgacat	pLL165, pLL205
I76C Rev	aaaggtgatcgctttgacgcatttgccgatgacagggat	pLL167, pLL153
K78C Rev	ttcaataaaggtgatcgcacagacgattttgccgatgac	pLL179, pLL206
I80C Rev	tttgatttcaataaaggtacacgctttgacgattttgcc	pLL193, pLL207
F82C Rev	ttcctctttgatttcaatacaggtgatcgctttgacgat	pLL184, pLL220
E84C Rev	gtctttttcctctttgatgcaaataaaggtgatcgcttt	pLL168, pLL155
A167C Rev	cggctttgtgtgtaggaagagcattgtgttgtttcaatattgcgccgg	pLL169
I76C I80K For	ggcaaatgcgtcaaagcgaagacctttattgaaatcaaa	pLL223
I76C I80K Rev	tttgatttcaataaaggtcttcgctttgacgcatttgcc	pLL223
Q65C For	aatatcaacccggacgccgcttgc gct atgtcaaagatccc	pLL215
M67C For	ccggacgccgctcaggcttgttcaaagatccctgtcatc	pLL216
K69C For	gccgctcaggctatgtcatgtatccctgtcatcggcaaa	pLL217
I70C For	gctcaggctatgtcaaagtgtcctgtcatcggcaaaatc	pLL218
V72C For	gctatgtcaaagatcccttgtatcggcaaaatcgtcaaa	pLL219
Q65C Rev	gggatctttgacatagcgcaagcggcgtccgggttgatatt	pLL215
M67C Rev	gatgacagggatctttgaacaagcctgagcggcgtccgg	pLL216
K69C Rev	tttgccgatgacagggatacatgacatagcctgagcggc	pLL217
I70C Rev	gattttgccgatgacaggacactttgacatagcctgagc	pLL218
V72C Rev	tttgacgattttgccgatacaagggatctttgacatagc	pLL219
